# *VaWRKY65* contributes to cold tolerance through dual regulation of soluble sugar accumulation and reactive oxygen species scavenging in *Vitis amurensis*

**DOI:** 10.1093/hr/uhae367

**Published:** 2025-01-03

**Authors:** Lin Meng, Huimin Zhou, Lisha Tan, Qingyun Li, Yujun Hou, Wenjuan Li, Subash Kafle, Ju Liang, Rishi Aryal, Zhenchang Liang, Haiping Xin

**Affiliations:** Key Laboratory of Plant Germplasm Enhancement and Specialty Agriculture, Wuhan Botanical Garden, Chinese Academy of Sciences, No. 201, Jiufeng 1st Road, Donghu New Technology Development Zone, Wuhan 430074, China; Key Laboratory of Plant Germplasm Enhancement and Specialty Agriculture, Wuhan Botanical Garden, Chinese Academy of Sciences, No. 201, Jiufeng 1st Road, Donghu New Technology Development Zone, Wuhan 430074, China; University of Chinese Academy of Sciences, No.19A, yuquan Road, Shijingshan Zone, Beijing 100049, China; Key Laboratory of Plant Germplasm Enhancement and Specialty Agriculture, Wuhan Botanical Garden, Chinese Academy of Sciences, No. 201, Jiufeng 1st Road, Donghu New Technology Development Zone, Wuhan 430074, China; University of Chinese Academy of Sciences, No.19A, yuquan Road, Shijingshan Zone, Beijing 100049, China; State Key Laboratory of Plant Diversity and Specialty Crops, Wuhan Botanical Garden, Chinese Academy of Sciences, No. 201, Jiufeng 1st Road, Donghu New Technology Development Zone, Wuhan 430074, China; Key Laboratory of Plant Germplasm Enhancement and Specialty Agriculture, Wuhan Botanical Garden, Chinese Academy of Sciences, No. 201, Jiufeng 1st Road, Donghu New Technology Development Zone, Wuhan 430074, China; University of Chinese Academy of Sciences, No.19A, yuquan Road, Shijingshan Zone, Beijing 100049, China; Key Laboratory of Plant Germplasm Enhancement and Specialty Agriculture, Wuhan Botanical Garden, Chinese Academy of Sciences, No. 201, Jiufeng 1st Road, Donghu New Technology Development Zone, Wuhan 430074, China; University of Chinese Academy of Sciences, No.19A, yuquan Road, Shijingshan Zone, Beijing 100049, China; Key Laboratory of Plant Germplasm Enhancement and Specialty Agriculture, Wuhan Botanical Garden, Chinese Academy of Sciences, No. 201, Jiufeng 1st Road, Donghu New Technology Development Zone, Wuhan 430074, China; University of Chinese Academy of Sciences, No.19A, yuquan Road, Shijingshan Zone, Beijing 100049, China; Turpan Institute of Agricultural Sciences, Xinjiang Academy of Agricultural Science, No. 845, munaer Road, Gaochang Zone, Turpan 838000, China; Department of Horticultural Science, North Carolina State University, 2721 Founders Drive, Raleigh, NC 27695, USA; Beijing Key Laboratory of Grape Science and Enology, and CAS Key Laboratory of Plant Resources, Institute of Botany, Chinese Academy of Science, No. 20, nanxincun, xiangshan, Haiding Zone, Beijing 100093, China; Key Laboratory of Plant Germplasm Enhancement and Specialty Agriculture, Wuhan Botanical Garden, Chinese Academy of Sciences, No. 201, Jiufeng 1st Road, Donghu New Technology Development Zone, Wuhan 430074, China

## Abstract

Although the significance of some plant WRKYs in response to cold stress have been identified, the molecular mechanisms of most WRKYs remain unclear in grapevine. In this study, we demonstrate that cold-induced expression of *VaBAM3* in *Vitis amurensis* executes a beneficial role in enhancing resistance by the regulating starch decomposition. VaWRKY65 was identified as an upstream transcriptional activator of *VaBAM3* through yeast one-hybrid library screening and validated to directly interact with the W-box region inside the *VaBAM3* promoter. Transgenic *Arabidopsis thaliana* plants and grapevine roots overexpression *VaWRKY65* exhibited improved cold tolerance along with higher BAM activity and soluble sugar levels, whereas opposite changes were observed in *VaWRKY65* knockdown lines created by virus-induced gene silencing (VIGS) in grapevine plants and in the knockout wrky65 mutants generated by CRISPR/Cas9 technology in grapevine roots. The transcriptome data show that overexpression of *VaWRKY65* led to significant alteration of a diverse set of stress-related genes at the transcriptional level. One of the genes, *Peroxidase 36* (*VaPOD36*), was further verified as a direct target of VaWRKY65. Consistently, *VaWRKY65*-overexpressing plants had higher *VaPOD36* transcript levels and POD activity but a reduced ROS level, while silencing *VaWRKY65* results in contrary changes. Collectively, these results reveal that VaWRKY65 enhanced cold tolerance through modulating soluble sugars produced from starch breakdown and ROS scavenging.

## Introduction

Grapevines are a highly cultivated and economically significant fruit crop worldwide. The grapevine industry encounters numerous environmental stresses, including biotic and abiotic stresses, which challenge its stable and sustainable development. It has been documented that extremely low temperatures have a negative impact on grapevine growth and development, as well as a reduction in fruit output and quality [1]. Most grapevine cultivars are originated from *Vitis vinifera*, which is particularly sensitive to cold stress [2, 3]. Consequently, the enhancement of grapevine cold resistance has constituted a principal pursuit for breeding endeavor, with the objective of averting the occurrence of severe cold damage during the winter season. Amur grapevine (*V. amurensis*), a cold-hardy wild grapevine species that can withstand −40°C in winter, has been widely used in traditional breeding to generate cold-tolerant cultivars [2]. However, improving grapevine through conventional cross-breeding poses several challenges because of a lengthy lifespan, the prevalence of inbreeding depression and the intricate genetic regulation of economic traits. Therefore, genetic engineering presents a potential novel approach to the breeding process. To facilitate the rapid and effective genetic engineering program, it is imperative to gain a fully comprehending of the genetic mechanisms and investigate valuable cold-responsive genes that have crucial functions in cold tolerance of grapevine.

Plants have undergone a complex evolutionary process, which has enabled them to adapt to and survive in rigorous environmental situations. Transcriptional remodeling of a range of downstream genes by transcription factors (TF) is an adaptive method to convert stress signals into cellular responses. Accumulating evidences have revealed that WRKYs perform regulatory functions in transcriptional networks by interacting with the W-box (TGAC) found inside the promoter of the downstream genes, thereby governing a wide array of biological events, including secondary metabolite synthesis, carbohydrate synthesis, ROS homeostasis, hormone signaling, and senescence [4–6]. Furthermore, WRKYs were also revealed to perform significant regulators in regulation of stress response, particularly cold response. For instance, *OsWRKY71* and *OsWRKY76* of rice function positively in cold resistance [7, 8], *AtWRKY34* acts a negative role in cold-induced mature pollen by suppressing CBF pathway gene expression [9]. In grapevine, *VaWRKY12* and *VaWRKY33* improved cold tolerance in grapevine callus [10, 11]. In bermudagrass, CdWRKY2 was found to regulate sucrose biosynthesis under cold stress [12]. *AtWRKY46* was reported to regulate osmotic stress responses by activating the antioxidant enzyme genes *MDHAR*, *GSTF14*, and *TRX5* [13]. Nevertheless, the precise function and underlying mechanism of *VaWRKYs* in cold tolerance remain elucidated.

The enrichment of various solute osmolytes has been demonstrated to significantly affect the ability of plants to withstand environmental stresses [14–20]. Soluble sugars are considered one of the most significant solute osmolytes, exerting a pivotal function in the attenuation of adverse consequences of stress by providing carbon and energy, stabilizing proteins and cell membranes, and modulating osmotic pressure [21, 22]. Previous investigations have demonstrated that leaf starch level decreases under cold conditions, providing a pool of soluble sugars [23, 24]. Many enzymes participate in producing and breaking down of soluble sugars, including β-amylases (BAM) [21, 25, 26]. BAMs are believed to facilitate the breakdown of starch into soluble sugars, which is crucial for accumulating soluble sugars during cold stress [27–29]. Furthermore, the soluble sugars resulting from starch degradation are transported from the chloroplasts to the cytoplasm and are involved in energy metabolism pathways to counteract cold stress [22, 30, 31]. In *Arabidopsis thaliana*, only BAM1 and BAM3 encode proteins that exhibit catalytic activity [29, 32, 33]. Several studies have reported that BAM activity and *BAM* gene expression levels are activated by cold stress, and *BAM* genes function positively in cold stress, suggesting the importance of BAM-mediated starch metabolic processes under cold [25, 34–37]. For example, *BAM3* mRNA levels were reported to be upregulated by low temperatures in *Arabidopsis*, kiwifruit, trifoliate orange, pear, and tea trees [33, 35–38]. The BAM activity was elevated more than fourfold in potato under cold stress [39]. Furthermore, the roles of *BAM* genes in stress tolerance have been extensively investigated. Diminished BAM activity and decreased cold tolerance are observed when *StBAM1* is silenced [40]. Overexpression of *AtBAM3* orthologues in several plant species results in enhanced soluble sugar content and increased freezing tolerance [35–37]. Even though these results highlight the significance of *BAMs* in cold tolerance, the transcriptional regulation of *BAM* genes and starch catabolism under cold stress remains largely unknown.

Cold stress can disrupt cellular homeostasis, leading to an elevation in the generation of ROS. The excessive concentrations of ROS are known to be detrimental to plant cells. Antioxidant enzymes are important roles in detoxifying ROS to combat environmental stresses [41, 42]. Many evidence has demonstrated that increased antioxidant activity or antioxidant gene expression could promote cold resistance by removing ROS in cucumber [43], citrus [44, 45], rice [46], and chickpea [47]. It has been suggested that soluble sugars may assist in the scavenging of ROS by providing fuel for NADPH-producing metabolic pathways. Furthermore, it has been demonstrated that stressful situations involving soluble sugar accumulation are associated with significant alterations in the balance of ROS [22, 48]. However, the correlation between antioxidant mechanism and carbohydrate metabolism, as well as upstream regulators of them, in cold stress remains unclear.

In our previous work, we established a transcriptome dataset and identified several vital genes and metabolites in a cold-hardy wild grapevine (*V. amurensis*) exposed to cold stress. We found that *VaBAM3* expression and soluble sugar content were upregulated by cold [49, 50]. Nevertheless, the role of *VaBAM3* in cold stress, as well as its upstream regulators are yet unknown. In this study, we discovered that *VaBAM3* positively contribute to cold tolerance through modulating soluble sugar accumulation. VaWRKY65 was fished out as an activator of *VaBAM3* and functions positively in cold tolerance through regulating soluble sugar accumulation. In addition, we also demonstrate that VaWRKY65 can modulate ROS homeostasis through activating *VaPOD36* transcript. Thus, our results indicate that *VaWRKY65* acts as a positive regulator in cold tolerance by regulating soluble sugar accumulation and ROS detoxification.

## Results

### Characterization and localization of *VaBAM3* protein

In previous work, we found that cold upregulated the transcript level of *VaBAM3* and increased soluble sugar levels [49, 50]. A phylogenetic tree and alignment analysis showed that VaBAM3 and AtBAM3 from *A. thaliana* belong to the same cluster and contains a catalytic and a binding site (Fig. S1A-B). Confocal imaging revealed that the VaBAM3 is a chloroplast protein.

### Cold induction of *VaBAM3*

**Figure 1:**
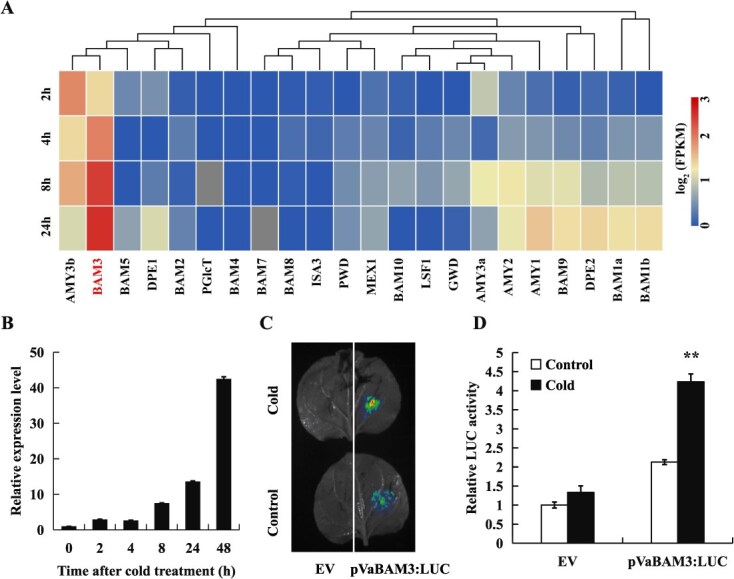
*VaBAM3* of grapevine (*V. amurensis*) is a cold-response gene. **A** Expression of genes involved in starch degradation and soluble sugar biosynthesis in cold-treated grapevine based on RNA-seq datasets. **B** Expression levels of *VaBAM3* under cold treatment, as calculated by quantitative real-time PCR. **C**-**D** LUC bioluminescence imaging (**C**) and relative LUC activity (**D**) in *N. benthamiana* leaves transformed with EV and pVaBAM3: LUC under low temperature. Error bars represent ± standard error (*n* = 3). Asterisks indicate significant difference based on the *t*-test (^**^P < 0.01)

To investigate the potential role of starch degradation and soluble sugar generation under cold condition, the expression of gene-related starch catabolism was identified (Table S2) [51]. Among them, *VaBAM3* was most strongly induced by cold in grapevine leaves according to *V. amurensis* transcriptome data under cold treatment (Fig. 1A). qRT-PCR analysis suggested that *VaBAM3* expression increased progressively under low-temperature treatment, peaking at 24 h (Fig. 1B). Then, the expressing the luciferase (LUC) reporter gene driven by *VaBAM3* promoter was transiently expressed in tobacco leaves assay under room temperature and cold stress. LUC fluorescence imaging and measurement showed that the LUC activity in leaves expressing *VaBAM3* promoter was significantly elevated with cold treatment (Fig. 1C and D). All these results suggest that *VaBAM3* is upregulated by cold treatment.

### *VaBAM3* functions positively in cold stress response

**Figure 2:**
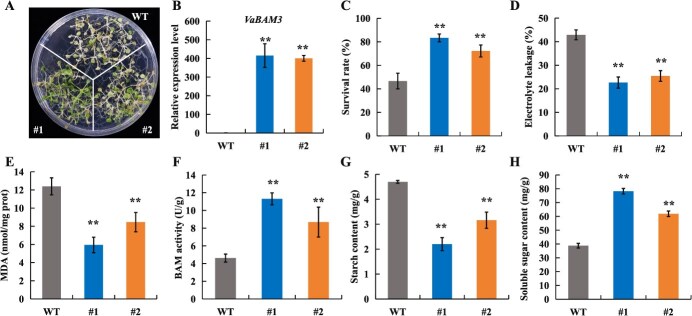
Overexpression of *VaBAM3* improves cold tolerance in *Arabidopsis*. **A** Phenotypes of *VaBAM3*-overexpression lines (#1 and #2) and wild-type (WT) after cold treatment. 2-week-old seedlings grown on 1/2 MS medium were subjected to −6°C for 1 h and transferred to 4°C for 12 h in the dark, followed by 3 days at 22°C. **B** Expression level of *VaBAM3* in WT and transgenic *Arabidopsis*. **C**-**E** Survival rate (**C**), EL (**D**), and MDA (**E**) contents in WT and transgenic *Arabidopsis* after cold treatment. **F**-**H** BAM activity (**F**), starch (**G**) and soluble sugar content (**H**) in WT and transgenic *Arabidopsis* after cold treatment. Error bars represent ± standard error (*n* = 3). Asterisks indicate significant difference based on the *t*-test (^*^P < 0.05, ^**^P < 0.01)

To explore the potential role of *VaBAM3* in cold response, two transgenic Arabidopsis lines overexpressing *VaBAM3* were generated (Fig. 2B). Following treatment fod 1 h at −6°C, wild-type (WT) plants displayed a more pronounced cold sensitivity relative to the transgenic lines (Fig. 2A). The cold-treated transgenic lines exhibited a notably increased average recovery rate in comparison with the WT following 3 d period at normal conditions (Fig. 2C). Remarkably reduced levels of electrolyte leakage (EL) and malondialdehyde (MDA) contents, which are important indicators of stress-induced damage, were observed in the *VaBAM3*-OE lines relative to WT after freezing treatment (Fig. 2D-E). Moreover, the BAM activities in *VaBAM3*-OE lines were markedly enhanced than those in WT (Fig. 2F). Consistent with this result, transgenic lines also displayed markedly reduced starch levels but higher soluble sugar contents than WT (Fig. 2G-H). Transgenic grapevine roots overexpressing *VaBAM3* were also produced by *Agrobacterium rhizogenes*-induced transformation system and subjected to a cold tolerance assay (Fig. S3A). While no distinction existed before low temperature treatment, transgenic roots exhibited much-reduced levels of EL and MDA relative to empty vector (EV) roots under cold stress (Fig. S3B-C). Similar to the transgenic *Arabidopsis* lines overexpressing *VaBAM3*, the transgenic grapevine roots also showed elevated BAM activity and soluble sugar content but reduced starch content compared to EV roots, especially under cold stress (Fig. S3D-F). In addition, staining with diaminobenzidine (DAB) and nitroblue tetrazolium (NBT) exhibited that the transgenic roots accumulated less H_2_O_2_ and O_2_^•-^ than the control under cold condition (Fig. S3G-H).

**Figure 3:**
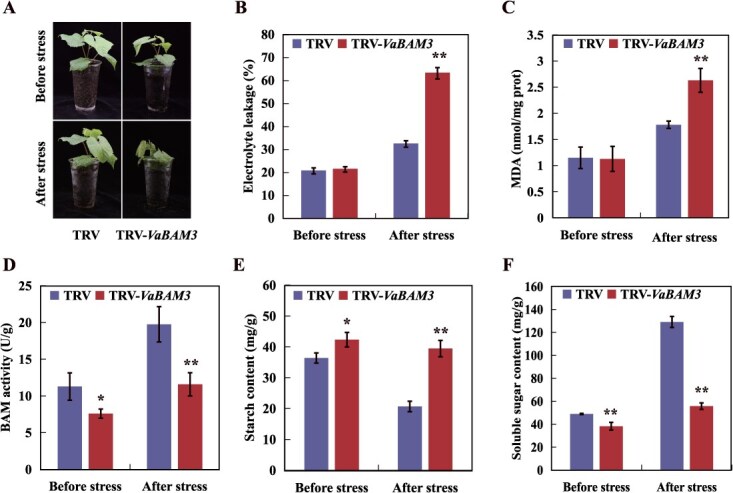
Silencing of *VaBAM3* decreases cold tolerance in grapevine. **A-C** Morphology (**A**), EL (**B**) and MDA levels (**C**) of TRV-*VaBAM3* and TRV control plant before and after cold treatment (−5°C for 6 h). D-F BAM activity (**D**), starch content (**E**) and soluble sugar content (**F**) measured before and after cold treatment (−5°C for 6 h) of TRV-*VaBAM3* and TRV control plant. Error bars represent ± standard error (*n* = 3). Asterisks indicate that the values are significantly different between the involved pairs (^*^P < 0.05, ^**^P < 0.01)

To further confirm the role of *VaBAM3* under cold stress response in grapevine, the native *VaBAM3* gene was suppressed by VIGS in *V. amurensis* seedling and knocked out in grapevine roots using CRISPR-Cas9. Genomic PCR and qRT-PCR were used to confirm the transformation and the down-regulation of *VaBAM3* expression (Fig. S4), meanwhile *bam3* mutant roots were verified using the Hi-TOM sequencing platform (Fig. S5A). After freezing treatment at −5°C for 6 h in VIGS plants and − 5°C for 4 h in grapevine roots, the TRV-*VaBAM3* exhibited more serious leaf wilting than TRV control plants (Fig. 3A). The TRV-*VaBAM3* and *bam3* roots exhibited significantly elevated levels of EL and MDA than TRV and EV control (Figs 3B-C and S5B-C). The TRV-*VaBAM3* plants and *bam3* roots showed a decrease in BAM activity and soluble sugar levels, but an increase in starch contents compared to TRV and EV control with and without the cold treatment (Figs 3D-F and S5D-F). Furthermore, the *bam3* roots showed markedly elevated concentrations of ROS in comparison to EV roots (S5G-H). These results suggest that *VaBAM3* positively contributes to cold tolerance by facilitating soluble sugar accumulation.

**Figure 4:**
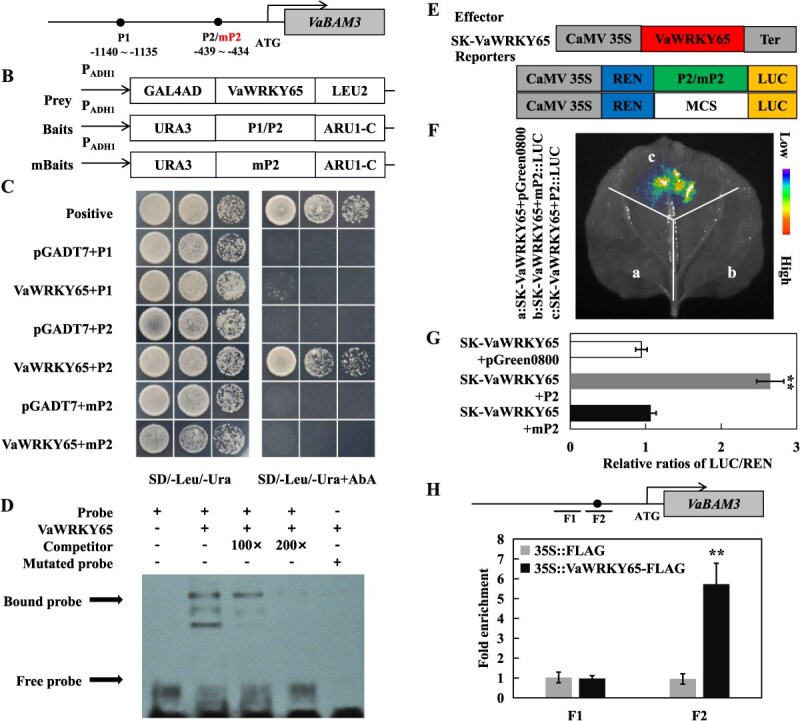
VaWRKY65 binds to and activates the promoter of *VaBAM3*. **A** Schematic diagrams of the promoters of *VaBAM3*. W-box elements were marked as black circles. P1 and P2 indicate truncated promoter sequences containing the W-box elements used for constructing baits. While mP2 are mutant versions that are changed from TGAC to TTTT. **B** The prey and bait vectors used for Y1H assays. **C** Y1H assays showing the binding of VaWRKY65 to promoters of *VaBAM3*. Positive control: p53-AbAi+pGAD-p53. All yeast cells grew on SD/−Leu/-Ura medium with (right) or without (left) AbA. **D** EMSA assay of the specific binding of VaWRKY65 to W-box elements in promoters of *VaBAM3*. The purified His-VaWRKY65 protein and biotin-labeled probe of designed fragments containing TGAC motif or mutated TTTT motif were used. Competitor was unlabeled probe at 100- and 200- fold. +: presence; −: absence. **E** Schematic diagrams of effector and reporter constructs used for dual-luciferase assay. **F** Live image of transcriptional activation of the *VaBAM3* promoter by VaWRKY65 in tobacco leaves using Dual-LUC system. **G** Quantitative analysis of dual-LUC transient expression assays of the promoter activity in tobacco protoplasts. Control is protoplasts co-transformed with the effector and the empty reporter vector, whose LUC/REN ratio was taken as 1 for normalization. **H** Enrichment of VaWRKY65 in the promoter of *VaBAM3*, revealed by ChIP-qPCR assays using specific primers designed for F1 and F2. Error bars represent ± standard error (*n* = 3). Asterisks demonstrate that the value is significantly different from that of the control (^**^P < 0.01)

### VaWRKY65 is a transcriptional activator of *VaBAM3*

To enhance comprehension of the regulation of *VaBAM3* under cold stress, we conducted Y1H-based screening to identify regulators of *VaBAM3* using the *VaBAM3* promoter fragment as bait. After sequencing all positive clones, VaWRKY65, a homolog of AtWRKY65, was identified by a constructed phylogenetic tree (Fig. S6A). The *VaWRKY65* expression was upregulated aggressively by cold (Fig. S6B). Microscopic observation indicated that VaWRKY65 is localized to the nucleus (Fig. S6C). Additionally, C-terminus was confirmed to serve as vital to the transcriptional activation of VaWRKY65 (Fig. S6D-E).

Bioinformatic motif search on the *VaBam3* promoter region identifies two W-boxes (Figs 4A and Fig. S12). To confirm the binding of VaWRKY65 and *VaBAM3* promoter, Y1H experiment was conducted first. The VaWRKY65 protein was used to act as prey, when *VaBAM3* promoter fragments P1 and P2 featuring W-box and one fragment (mP2) containing modified W-box were employed to create baits (Fig. 4A and B). Yeast grew successfully on selective media. However, only yeast cells harboring VaWRKY65 prey protein and P2 bait were able to survive when treated with Aureobasidin A (AbA; 200 ng/ml) (Fig. 4C), suggesting that VaWRKY65 could bind to P2 element in *VaBAM3* promoter. We then carried out electrophoretic mobility shift assay (EMSA) to test the binding of VaWRKY65 to the W-box in P2. The EMSA results showed that His-VaWRKY65 protein could bind to the probe containing W-box of P2. However, presence of the competitor DNA reduced binding capacity in a dosage-dependent manner, and the binding band shift was eliminated with mutant probe (Fig. 4D). Further investigation was conducted using a dual LUC reporter assay to examine the *in vivo* regulation of *VaBAM3* expression by VaWRKY65 (Fig. 4E). Co-infiltration with VaWRKY65-SK effector and *VaBAM3* reporter led to a substantial enhancement in LUC fluorescence relative to the control, whereas mutation of W-box entirely suppressed the LUC activity (Fig. 4F). Additional validation of the LUC fluorescence imaging was further supported by quantitative measurement LUC/REN ratios (Fig. 4G). The chromatin immunoprecipitation (ChIP)-qPCR results showed that VaWRKY65 was significantly occupied by F2 fragment containing W-box in *VaBAM3* promoter, but not in other fragments without W-box (Fig. 4H). These results reveal that VaWRKY65 could target the W-box in *VaBAM3* promoter to activate its expression.

### *VaWRKY65* functions positively during cold stress response and regulates starch degradation

**Figure 5:**
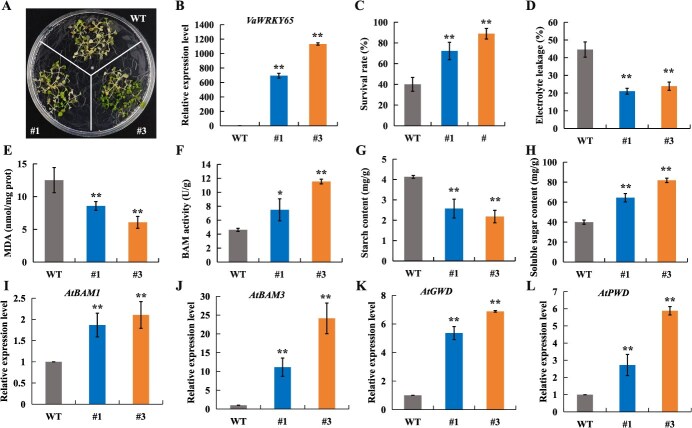
Overexpression of *VaWRKY65* improves cold tolerance in *Arabidopsis*. **A** Phenotypes of *VaWRKY65*-overexpression lines (#1 and #3 and wild-type (WT) after cold treatment. 2-week-old seedlings grown on 1/2 MS medium were subjected to −6°C for 1 h and transferred to 4°C for 12 h in the dark, followed by 3 days at 22°C. **B** qRT-PCR analysis verified the expression levels of *VaWRKY65* in WT and transgenic *Arabidopsis*. **C**-**E** Survival rate (**C**), EL (**D**), and MDA (**E**) contents in WT and transgenic *Arabidopsis* after cold treatment. **F**-**H** BAM activity (**F**), starch content (**G**) and soluble sugar content (**H**) in WT and transgenic *Arabidopsis* after cold treatment. **I**-**L** Expression levels of *AtBMA1* (**I**), *AtBAM3* (**J**), *AtGWD* (**K**) and *AtPWD* (**L**) in the WT and two transgenic lines (#1 and #3). Error bars represent ± SE (*n* = 3). Asterisks indicate significant difference between WT and transgenic *Arabidopsis* (^*^P < 0.05, ^**^P < 0.01)

Given the fact that VaWRKY65 is upregulated by cold treatment (Fig. S6B) and acts as an upstream regulatory protein of *VaBAM3* (Fig. 4), we predicted that VaWRKY65 may contribute to cold tolerance via controlling starch decomposition. To verify this hypothesis, *VaWRKY65* was overexpressed in *Arabidopsis* to generate transgenic plants for cold tolerance assays (Fig. 5A and B). After being exposed to −6°C for 1 h, the *VaWRKY65*-OE transgenic lines showed increased cold tolerance with increased survival rates compared to WT (Fig. 5A and C). The *VaWRKY65*-OE lines exhibited notably reduced EL and MDA content compared to WT when subjected to cold treatment (Fig. 5D and E). After freezing treatment, the BAM activity levels in the *VaWRKY65*-OE lines were dramatically elevated relative to WT (Fig. 5F). In comparison to WT, the *VaWRKY65*-OE lines showed a reduction in starch content and an increase in soluble sugars. (Fig. 5G and H). Moreover, the expressions of starch degradation-related genes (*AtBAM1*, *AtBAM3*, *AtGWD,* and *AtPWD*) were markedly up-regulated in *VaWRKY65*-OE compared to WT (Fig. 5I-L).

For an expanded comprehension of how VaWRKY65 regulates cold tolerance and starch decomposition in grapevine, transgenic grapevine roots overexpressing *VaWRKY65* were generated (Fig. S7A). A significantly increased transcription level of *VaBAM3* was observed in transgenic grapevine roots (Fig. S7B). After exposure to −5°C for 4 h, EL and MDA levels in *VaWRKY65*-OE lines were markedly reduced compared to EV roots control (Fig. S7C-F). Moreover, cold treatment led to a notable reduction in starch content and a greater elevation in soluble sugar level and BAM activity in *VaWRKY65*-OE transgenic lines (Fig. S7G-I).

**Figure 6:**
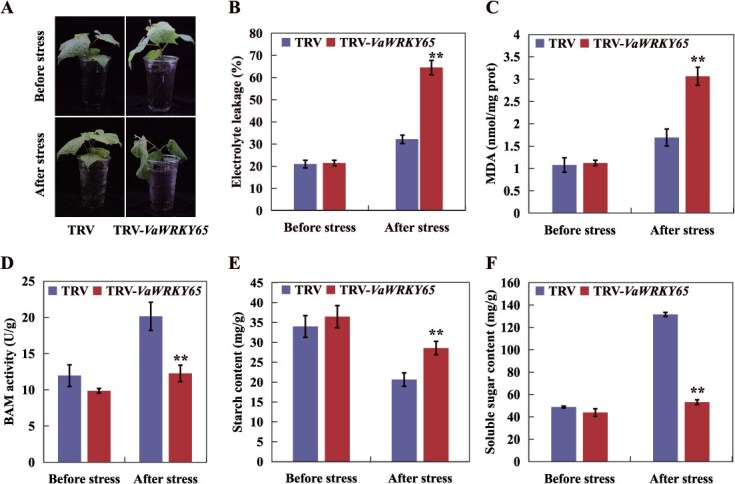
Silencing of *VaWRKY65* decreases cold tolerance in *V. amurensis*. **A-C** Morphology, EL (**B**) and MDA (**C**) levels of TRV-*VaWRKY65* and TRV control plant before and after cold treatment (−5°C for 6 h). **D-F** BAM activity (**D**), starch content (**E**) and soluble sugar content (**F**) measured before and after cold treatment of TRV-*VaWRKY65* and TRV control plant. Error bars represent ± standard error (*n* = 3). Asterisks indicate that the values are significantly different between the involved pairs (^*^P < 0.05, ^**^P < 0.01)

To further address the function of *VaWRKY65* in grapevine cold tolerance, we silenced *VaWRKY65* in the seedlings and roots of grapevine using VIGS and CRISPR-Cas9-mediated knockout, respectively. The TRV-*VaWRKY65* were verified using qRT-PCR and genomic PCR (Fig. S8A and B), while the editing in *wrky65* grapevine root genomes was detected by the Hi-TOM sequencing platform as mentioned above (Fig. S9A). The *VaBAM3* expression was significantly repressed in the VIGS line and *wrky65* roots compared with the control (Figs S8C and S9B). In the presence of cold treatment (−5°C for 6 h), TRV-*VaWRKY65* plants showed more serious leaf damage compared to TRV plants (Fig. 6A). EL and MDA contents were at same level between the VIGS lines or *wrky65* grapevine roots and the TRV or EV control under room temperature. Nevertheless, when subjected to cold treatment, the VIGS line and *wrky65* grapevine roots exhibited notably elevated levels of EL and MDA in comparison with control (Figs 6B-C and S9C-D). Additionally, the VIGS line and *wrky65* grapevine roots displayed a higher starch content but lower soluble sugar content and BAM activity relative to TRV and EV control, both with and without the cold treatment (Figs 6D-F and S9G-I). These results indicate that *VaWRKY65* functions positively in cold tolerance via modulating BAM3-mediated starch breakdown.

### Overexpression of *VaWRKY65* results in remarkable reprogramming

**Figure 7:**
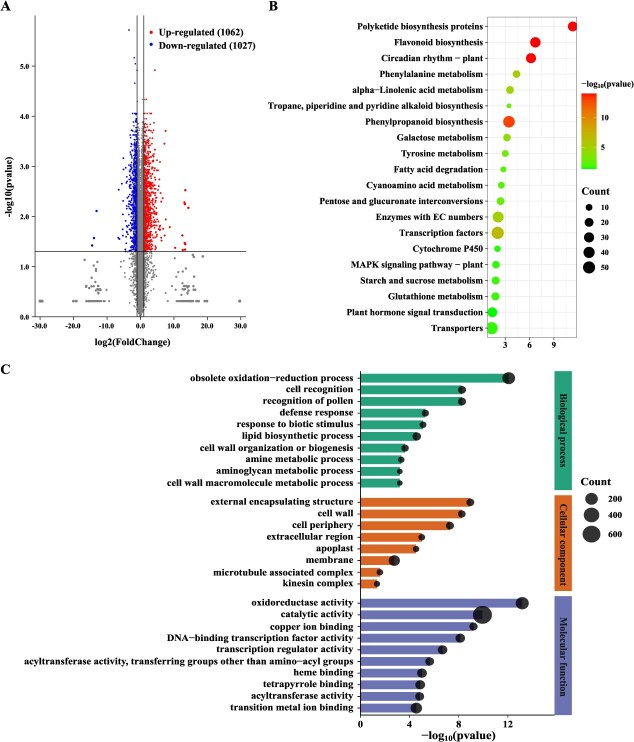
Overexpression of *VaWRKY65* leads to transcriptional reprogramming in transgenic grapevine roots. **A** Volcano plot of DEGs in comparison of EV and *VaWRKY65*-OE transgenic grapevine roots. **B-C** KEGG (**B**) and GO (**C**) enriched analyses between EV and *VaWRKY65*-OE transgenic grapevine roots

To gain a deeper comprehension of VaWRKY65-mediated networks, RNA-seq was conducted using EV control roots and transgenic grapevine roots (Table S3). To detect differentially expressed genes (DEGs), a criterion of a fold change more than or equal to 2 and a significance level at least 0.05 were used, 2089 (1062 up and 1027 down) DEGs were observed in transgenic grapevine roots compared with EV roots (Fig. 7A). Among them, *VaBAM3 (VIT_202s0012g00170)* was significantly upregulated in the transgenic roots as we expected (8.9-fold of control, Table S4).

The KEGG analysis revealed that the most enriched KEGG pathways were associated with flavonoids, phenylalanine, starch and sucrose, galactose and glutathione (Fig. 7B), suggesting that VaWRKY65 may regulate a wide range of metabolic pathways to enhance the cold tolerance. Of note, KEGG enrichment in starch and sucrose metabolism pathway supported aforementioned results. Moreover, GO analysis showed that DEGs associated with biological processes were mostly linked to biosynthetic process, iron ion homeostasis, recognition of pollen, lignin catabolic process, and cinnamic acid biosynthetic process. For cellular component, DEGs were predominantly associated with extracellular region, apoplast, cell wall, and plasma membrane. For molecular functions, the GO terms were enriched in copper ion binding, ferroxidase activity, oxygen oxidoreductase activity, oxidoreductase activity, and peroxidase activity, which implies the ROS scavenging-related pathways might be activated in *VaWRKY65* overexpression lines. (Fig. 7C). Collectively, these results demonstrated that, besides *VaBAM3*-mediated starch degradation, overexpression of *VaWRKY65* caused a considerable alteration in transcription of genes responsible for many metabolic pathways.

### VaWRKY65 binds to promoter of *VaPOD36* to activate its transcription

**Figure 8:**
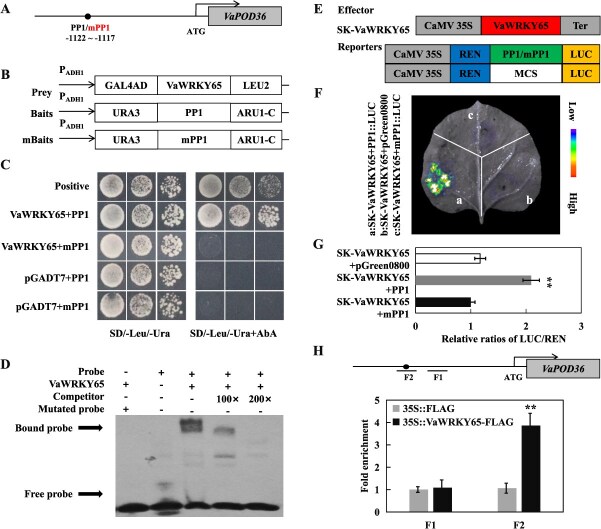
VaWRKY65 binds to and activates the promoter of *VaPOD36*. **A** Schematic diagrams of the promoters of *VaPOD36*. W-box elements were marked as black circles. PP1 indicate truncated promoter sequence containing the W-box element used for constructing bait. While mPP1 are mutant versions which are changed from TGAC to TTTT. **B** The prey and bait vectors used for Y1H assays. **C** Y1H assays showing the binding of VaWRKY65 to promoters of *VaPOD36*. Positive control: p53-AbAi+pGAD-p53. All yeast cells grew on SD/−Leu/-Ura medium with (right) or without (left) AbA. **D** EMSA assay of the specific binding of VaWRKY65 to W-box elements in promoters of *VaPOD36*. The purified His-VaWRKY65 protein and biotin-labeled probe of designed fragments containing TGAC motif or mutated TTTT motif were used. Competitor was unlabeled probe at 100- and 200-fold. +: presence; −: absence. **E** Schematic diagrams of effector and reporter constructs used for dual-luciferase assay. **F** Live image of transcriptional activation of the *VaPOD36* promoter by VaWRKY65 in tobacco leaves using Dual-LUC system. **G** Quantitative analysis of dual-LUC transient expression assays of the promoter activity in tobacco protoplasts. Control is protoplasts co-transformed with the effector and the empty reporter vector, whose LUC/REN ratio was taken as 1 for normalization. **H** Enrichment of VaWRKY65 in the promoter of *VaPOD36*, revealed by ChIP-qPCR assays using specific primers designed for F1 and F2. Error bars, ± SE (*n* = 3). Asterisks demonstrate that the value is significantly different from that of the control (^**^P < 0.01)

Since the GO analysis of molecular functions suggested that ROS scavenging-related pathways might be activated in *VaWRKY65* overexpression lines, *VaPOD36* expression (*VIT_204s0023g02570*) of the enriched GO term “peroxidase activity” was notably increased in the transgenic line. Promoter sequence analysis showed that the *VaPOD36* promoter harbors a W-box motif, a potential binding site for VaWRKY65 TF (Figs 8A and S12). The Y1H experiment was performed to confirm the interaction between VaWRKY65 and *VaPOD36* promoter, using all constructed prey and bait vectors (Fig. 8B). As showed in Fig. 8C, yeast cells harboring VaWRKY65-AD and PP1 exhibited robust growth on media supplemented with AbA (150 ng/ml), mutation of the TGAC to TTTT entirely suppressed the yeast growth (Fig. 8C). EMSA analysis revealed that His-VaWRKY65 exhibited binding affinity towards the W-box-containing DNA probe, which was then efficiently competed out by 100-fold and 200-fold excess of unlabeled DNA competitor, respectively. Conversely, the binding band was absent when the W-box was mutated (Fig. 8D). Further investigation was carried out using a dual LUC reporter assay to examine the *in vivo* regulation of *VaPOD36* expression by VaWRKY65. The results of the LUC fluorescence analysis and quantitative measurement indicated that co-expression of SK-VaWRKY65 effector and original *VaPOD36* promoter reporter resulted in an increase in LUC activity in comparison to the control. However, mutation of W-box led to the suppression of the LUC activity (Fig. 8E-G). In the ChIP-qPCR assay, the F2 fragment containing W-box in *VaPOD36* promoter was significantly enriched by VaWRKY65 (Fig. 8H). These results reveal that VaWRKY65 activates *VaPOD36* transcription through interacting with the W-box inside its promoter. And the upregulation of *VaPOD36* by VaWRKY65 can result in elevated POD enzyme activity. The enhanced antioxidant capacity may facilitate the decomposition of hydrogen peroxide into water and oxygen, which in turn reduces ROS accumulation in cells and enhances the capacity of plants to tolerate cold stress.

### POD activity, *VaPOD36* expression and ROS accumulation in *VaWRKY65* transgenic and VIGS lines

**Figure 9:**
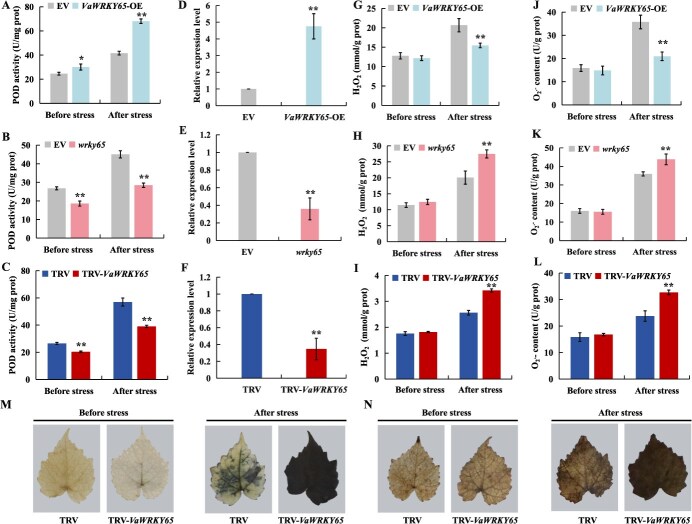
VaWRK65 regulates *VaPOD36* expression to modulate ROS accumulation under cold stress. **A-L** POD activity (**A**-**C**), *VaPOD36* expression (**D**-**F**), H_2_O_2_ (**G**-**I**) and O_2_^•-^ (**J**-**L**) levels in the different lines before and after cold treatment. **M**-**N** NBT (**M**) and DAB (**N**) in leaves from TRV-*VaWRK65* and TRV control plants with cold treatment. For transgenic grapevine roots, 2-week-old roots were treated at −5°C for 4 h and transferred to 4°C for 12 h in the dark. For VIGS plants, two-month-old VIGS seedlings were subjected to −5°C for 6 h. Error bars represent ± standard error (*n* = 3). Asterisks indicate significant differences between different groups under the same growth condition (^*^P < 0.05, ^**^P < 0.01)

Given that *VaPOD36* is directly targeted by VaWRKY65, POD activity and *VaPOD36* expression were measured in transgenic and mutant roots and VIGS lines. In transgenic roots overexpressing *VaWRKY65*, the POD enzyme activity was dramatically elevated, especially when subjected to cold stress. Conversely, in *wrky65* roots and the VIGS line displayed a reduce POD enzyme activity (Fig. 9A-C). Additionally, elevated levels of *VaPOD36* were observed when *VaWRKY65* was overexpressed, while diminished levels were noted when *VaWRKY65* was silenced (Fig. 9D-F). The results demonstrate that overexpression of *VaWRKY65* enhanced POD activity and *VaPOD36* expression, whereas the knockdown of *VaWRKY65* resulted in a decline in these parameters.

The role of POD in scavenging ROS is well established, thus we analyzed the level of H_2_O_2_ and O_2_^•-^ in transgenetic plants. Under cold treatment, the *VaWRKY65*-overexpressing line showed markedly reduced concentrations of H_2_O_2_ and O_2_^•-^ relative to the control. By contrast, H_2_O_2_ and O_2_^•-^ concentrations were notably elevated in *wrky65* roots and VIGS lines compared with the control (Fig. 9G-L). Histological examination using DAB and NBT revealed that leaves from the VIGS line and roots from *wrky65* exhibited deeper staining compared to the control after low temperature treatment (Figs 9M-N and Fig. S9E-F), and roots of *VaWRKY65*-overexpressing lines represented lighter staining than the control (Fig. S7E-F). These results illustrated that overexpression of *VaWRKY65* significantly reduces the accumulation of ROS while silencing *VaWRKY65* elevates ROS accumulation.

### Silencing of *VaPOD36* increases cold sensitivity in grapevine

**Figure 10:**
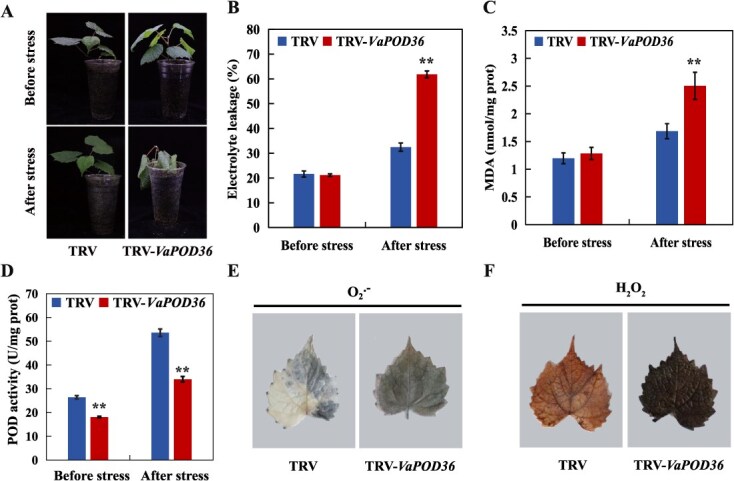
Silencing of *VaPOD36* decreases cold tolerance in *V. amurensis*. **A-D** Morphology (**A**), EL (**B**), MDA (**C**) levels and POD activity (**D**) of TRV-*VaPOD36* and TRV control plant before and after cold treatment (−5°C for 6 h). **E-F** NBT (**E**) and DAB (**F**) in leaves from TRV-*VaPOD36* and TRV control plants with cold treatment. Error bars represent ± standard error (*n* = 3). Asterisks indicate significant differences between different groups under the same growth condition (^*^P < 0.05, ^**^P < 0.01)

To further confirm the function of *VaPOD36* in clod response, *VaPOD36* was silenced in *V. amurensis* seedlings by VIGS method. Genomic PCR and qRT-PCR were used to confirm the transformation and the down-regulation of *VaPOD36* expression (Fig. S10). In the presence of clod treatment at −5°C for 6 h, the TRV-*VaPOD36* displayed more serious leaf damage than TRV control plants (Fig. 10A). The levels of EL and MDA were elevated in the TRV-*VaPOD36* than TRV control (Fig. 10B and C). In addition, the POD activity was dramatically decrease in TRV-*VaPOD36* (Fig. 10D). Meanwhile, the TRV-*VaPOD36* plants accumulated more O2^•-^ and H_2_O_2_ than the TRV control under cold stress (Fig. 10E). These results revealed that silencing of *VaPOD36* dramatically reduces the cold tolerance of grapevine.

**Figure 11:**
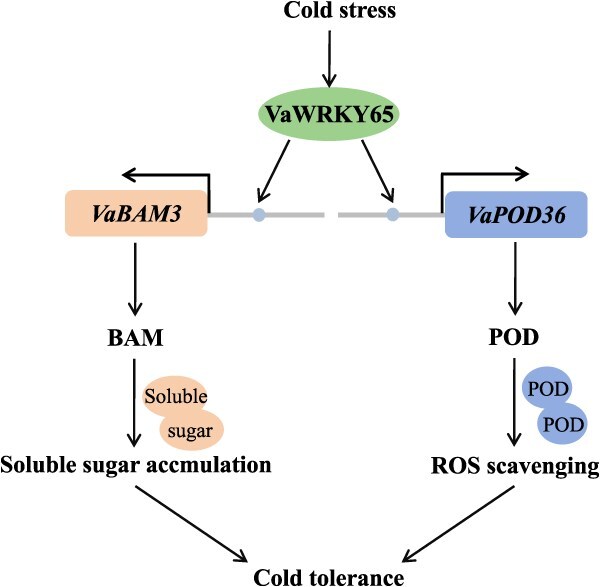
A proposed working model illustrating the regulatory network of VaWRKY65 in cold tolerance. VaWRKY65 is induced by cold stress, which subsequently activates *VaBAM3* and *VaPOD36* transcription via interacting with the W-box in their promoters to improve plant starch degradation and ROS scavenging captivity, leading to increased plant cold tolerance

## Discussion

Living organisms have developed various molecular processes to cope with changing circumstances, especially clod stress [24]. Many studies have revealed that TF families, including WRKY TF family, play a pivotal role in enabling plants to cope with the detrimental effects of cold stress [5, 52]. Nevertheless, it is still not clear what role WRKYs play in the physiological and molecular responses to cold stress, particularly in perennial plants such as grapevine. Our results demonstrate that VaWRKY65 functions positively in cold tolerance by obtaining a dual regulation of soluble sugar accumulation and ROS detoxification via transcriptionally regulating *VaBAM3* and *VaPOD36.*

Although plant WRKYs were documented to be instrumental in regulating cold response, the underlying physiological mechanisms are still poorly elucidated. In tomato, *SlWRKY33* functions positively in enhancing cold resistance through targeting and inducing *SlCDPK11*, *SlMYBS,* and *SlBAG6* [53]. CsWRKY46 from cucumber activates the expression of *CsABI5*, which confers cold tolerance in dependent on ABA signaling [54]. Our results demonstrated that *VaWRKY65* plays a positive role in cold tolerance by modulating starch degradation and ROS homeostasis by regulating *VaBAM3* and *VaPOD36*. Our work offers novel explanations for the molecular function and mechanism involved in the cold tolerance response mediated by VaWRKY65 and offers a beneficial source of genetic material that can be utilized in genetic engineering techniques to enhance cold tolerance. Additionally, transcriptome analysis revealed that overexpression of *VaWRKY65* causes comprehensive transcriptional alteration (Fig. 7). In addition, transcriptome data showed that overexpression of *VaWRKY65* exerted a significant impact on several pivotal metabolic pathways, including those involved in flavonoid, phenylpropanoid, starch, and sucrose metabolism via the regulation of relevant genes. Flavonoids are plant-specific metabolites with multiple important functions, including conferring color to flowers to attract pollinators and acting as antioxidants that regulate levels of ROS to control plant growth, development, and fertility [55]. The TRV-*VaWRKY65* plants showed decreased proanthocyanidin and total flavonoid contents compared to TRV (Fig. S11). The upregulation of the flavonoid biosynthesis may contribute to protecting plants from oxidative stress induced by cold stress. It is noteworthy that the transgenic line also exhibits a notable enrichment in phenylpropanoid biosynthesis. The PAL (phenylalanine ammonia-lyase) represents a rate-limiting enzyme in the phenylpropanoid pathway, which is associated with the synthesis of a range of secondary metabolites, including flavonoids, lignin, stilbenes, and anthocyanins. These metabolites have been demonstrated to serve a protective function in the coping with abiotic stresses [56]. Moreover, galaetose metabolism and starch and sucrose metabolism are also enriched in the transcriptome. The degradation of starch into soluble sugars under cold stress may increase the cellular energy supply to maintain cellular physiological activities and repair damage caused by cold stress [22, 23]. Thus, it can be hypothesized that *VaWRKY65* plays a role in regulating the biosynthesis of these metabolites, either directly or indirectly, which in turn may impart cold tolerance. Nevertheless, further research is necessary to substantiate this hypothesis in the future. The mitogen-activated protein kinase (MAPK) signaling cascades facilitate the transfer of signals from the receptor to the TF via a series of phosphorylation actions [57, 58]. A conserved region in the N-terminus of WRKY can be activated by MAPK-dependent phosphorylation [57, 59]. Herein, we found the MAPK signaling pathway was also identified as a notably enriched KEGG pathway, implying that VaWRKY65 may be phosphorylated by some MPK kinase proteins involved in MAPK cascades to positively regulate clod response. Additionally, our analysis revealed a substantial upregulation of genes correlated with a spectrum of metabolic pathways, suggesting that *VaWRKY65* could enhance cold resistance via regulating other diverse metabolites including flavonoid, phenylalanine, and glutathione (Fig. 7A and B). Therefore, other potential pathways regulated by VaWRKY65 need more extra work to investigate.

Starch is believed to confer beneficial effects on enhancing stress tolerance. Plants break down starch to release soluble sugars to counteract the effects of harsh environmental conditions [60–63]. It has reported that soluble sugars serve as osmoprotectants in response to stress. Accumulation of soluble sugars under conditions of stress reduces the water potential of the cell, thereby enabling the retention of water and the maintenance of cell turgor without affecting normal metabolic processes to survive under stressful conditions [24]. In the presence of adverse conditions, the process of photosynthesis in plants may be impeded, leading to an insufficient supply of energy. The soluble sugars produced through the degradation of starch can provide the energy to sustain fundamental cellular metabolic processes under stresses [22]. Under osmotic stress, such as drought or cold, the accumulation of soluble sugars can help maintain cell membrane stability. This is because sugars can replace water molecules around membrane lipids and proteins, preventing the loss of membrane integrity and protein denaturation that can occur during dehydration [64, 65]. Research conducted on *Arabidopsis* mutants has shown that *GWD* and *BAM3* were upregulated and serve as the principal catalysts participated in degradation of starch in cold condition [66, 67]. In our study, *VaBAM3* has the closest relationship to *AtBAM3* based on multiple alignments (Fig. S1). The transcript level of *VaBAM3* and the activation of *VaBAM3* promoter were dramatically induced by cold conditions in *V. amurensis*, implying that *VaBAM3* may function positively in starch degradation under low-temperature conditions (Fig. 1). It is important to note that of the BAM family members, only *AtBAM3* has been reported to identified as a vital component starch degradation and is upregulated by cold [29, 33]. In this study, overexpression of *VaBAM3* led to enhanced cold resistance, but the knockout of *VaBAM3* profoundly decreased cold tolerance. It is worth noting that sugar accumulation and starch degradation positively correlated with *VaBAM3* expression under cold stress (Figs 2-3, S3, and S4). These findings suggest that *VaBAM3* participates in starch degradation and enhances cold tolerance. Moreover, the homolog genes of *AtBAM3* in other plant species, such as trifoliate orange, kiwifruit, tea, and pear, were also reported to function positively under cold stress and starch degradation [33, 35–38]. These results imply the conserved function of *BAM3* during cold stress response in plants and thus provide potential genes that may be utilized for enhancing the cold tolerance of crops through genetic enhancement.

The BAM gene encodes an enzyme with catalytic activity and directly contributes to mitigating cold damage by generating soluble sugars [29, 33, 68]. Several TFs have been documented to interact with the promoters of genes associated with soluble sugar synthesis, either promoting or suppressing their expression, thereby modulating starch metabolism, which is critical for plant stress response [17, 29, 35, 37, 69, 70]. However, the TFs that govern BAM-mediated starch catabolism in cold response in fruit crops remain characterized. It is noteworthy that ABF-, NAC-, MYB-, and MYC- binding elements were identified in *VaBAM3* (Fig. S12), indicating that additional upstream regulators might be involved in modulating *VaBAM3*-mediated starch degradation during cold stress in grapevine. In kiwifruit, AaCBF4 was found to enhance freezing tolerance by directly activating *AaBAM3.1* expression [37]. PtrABF4 and PtrABR1 interact directly with the promoter of *PtrBAM3* to regulate starch catabolism under drought stress [17]. In our study, *VaWRKY65* was fished out as the upstream positive regulator in *VaBAM3*-mediated cold response in grapevine (Figs 4-6 and Figs S7-9). Therefore, it is worthwhile to further investigate the transcriptional modules formed by TFs, such as WRKY, CBF, ABF, and other TFs, to orchestrate various biological physiological and processes by targeting downstream genes. Additionally, soluble sugar is important compound controlling both fruit flavor and plant stress tolerance. The molecular mechanism underlying soluble sugar accumulation in grapevine fruit is also needed to be systematically studied. However, we found that *VaWRKY65* is not highly expressed in grapevine fruit, even under cold condition [71]. Therefore, further investigation is required to elucidate the potential roles of other crucial genes involved in regulating soluble sugar accumulation in grapevine fruits.

Abiotic stress can cause high levels of ROS, which are toxic to plant cells and can result in damage to nucleic acids, proteins, and lipid peroxidation, ultimately leading to cell death [41]. Therefore, preserving the balance of ROS levels and antioxidant metabolism is critical for plants to combat stressful environments. Plants possess a complex ROS scavenging system including enzymatic antioxidants, such as POD, CAT, SOD, and GST, to cope with abiotic stresses, including cold [41, 72, 73]. In this study, we found that ROS level was significantly decreased in the transgenic lines overexpressing *VaWRKY65*, whereas the *VaWRKY65*-knockout and VIGS plants accumulated noticeably more ROS after cold treatment compared to WT or control plants (Figs 9G-N, S7E-F, and S9E-F). Following these alterations, the activities of POD and an H_2_O_2_ scavenging-related gene, *VaPOD36*, were upregulated in *VaWRKY65* overexpressed plants and downregulated in the mutant and silenced line, especially under cold stress (Fig. 9A-F). This suggests that the ROS-eliminating ability was improved when *VaWRKY65* was overexpressed but hindered when *VaWRKY65* was silenced. Furthermore, VaWRKY65 can bind to the W-box within *VaPOD36* promoter to activate its expression (Fig. 8). In other works, AtWRKY46 was reported to activate the antioxidative enzyme gene *AtPOD54* by binding to its promoter to positively regulate dehydration stress [13]. MsWRKY33 improves alfalfa tolerance to salt stress by activating *MsERF5* expression to regulate ROS homeostasis [74]. Overall, this result enabled us to establish a new regulatory module consisting of VaWRKY65-*VaPOD36* to orchestrate ROS balance in cold response. Furthermore, it would be beneficial to consider the long-term physiological effects of reduced ROS accumulation under cold stress. The reduction of ROS through VaWRKY65 regulation may potentially lead to improved cellular homeostasis under cold stress, thereby promoting overall plant growth. This is supported by the understanding that ROS can cause oxidative damage to cellular components, including cellular membranes, proteins and nucleic acids, which can inhibit growth [73, 74]. By maintaining lower ROS levels, plants can allocate more resources to growth processes rather than repair mechanisms. Stress conditions that lead to high ROS levels can negatively impact reproductive structures and processes, such as pollen viability and seed set [75, 76]. By mitigating ROS accumulation, *VaWRKY65* may help protect these sensitive reproductive tissues, thus potentially enhancing fertility under stress.

It has been demonstrated that soluble sugars possess antioxidant properties, which can effectively counteract oxidative stress triggered by ROS [31, 65]. Interestingly, our results revealed that ROS levels were markedly diminished in *VaBAM3*-OE lines with higher soluble sugar content, ROS levels were noticeably increased in the *bam3* lines with decreased soluble sugar content (Figs S3G-H and S5G-H). While ROS can be generated in other parts of the cell, the chloroplast is where most ROS are produced [76–78]. Notably, VaBAM3 is localized in the chloroplast (Fig. S2). Thus, we hypothesize that overexpression of *VaBAM3* resulted in elevated level of soluble sugars and its metabolites in the chloroplast, whereby these compounds may act either independently or in conjunction with other antioxidants to remove ROS generated within this organelle. Additionally, soluble carbohydrates can reduce ROS levels in cytosol [48]. Therefore, the diffusion of ROS from their initial location to other cellular regions may be substantially reduced, leading to a notable decrease in ROS accumulation, as evidenced by the significant reduction in ROS levels observed in *VaBAM3*-overexpressing lines. Nevertheless, the precise manner in which soluble sugars interact with ROS signaling pathways in plant cold response remains poorly understood. Further study is needed to uncover the role of central members that simultaneously regulate soluble sugars and ROS homeostasis to elucidate the cold tolerance mechanism and facilitate the enhancement of cold tolerance.

Taken together, we propose a working model to elucidate how a cold-induced WRKY member *VaWRKY65* functions in response to cold stress (Fig. 10). VaWRKY65 is shown to have a dual function in modulating both starch degradation and ROS scavenging in cold response. VaWRKY65 could activate the starch degradation gene (*VaBAM3*) and the POD-encoding gene (*VaPOD36*) expressions via binding to the W-box in their promoter, which leads to more soluble sugar accumulation and a decrease in ROS accumulation to enhance cold tolerance in grapevine. The research provides new insights into the molecular mechanism responsible for carbohydrate distribution in plant responses to cold stress. However, further research is needed to explore the up-regulator of *VaWRKY65* and the correlation between the antioxidant system and carbohydrate metabolism. Additionally, the lack of whole-plant grapevine experiments under cold stress is still a limitation for functional validation, and thus a continued necessity for further whole-plant grapevine transformation efforts to be undertaken in order to complete our research.

## Materials and methods

### Plant materials and growth conditions

The grapevine micropropagates were cultivated on 1/2 MS (pH 5.8) and maintained in a growth chamber set at 25°C with 16 h of light and 8 h of darkness. For analysis of gene expression, two-month-old seedlings were incubated at 4°C, and the leaves were harvested at 0, 2, 4, 8, 24, and 48 h. The collected leaves were frozen rapidly using liquid nitrogen and then stored at −80°C for further analysis.

### RNA extraction and qRT-PCR analysis

Total RNA was extracted from grapevine and *Arabidopsis* leaves and roots using a commercially available RNA purification kit (TIANGEN, Beijing, China). The first-strand cDNA was obtained following the instructions provided in the First Strand cDNA Synthesis Kit (Transgen, Beijing, China). qRT-PCR was conducted on a QuantStudio 7 Flex PCR machine (Thermo Fisher Scientific, Inc., Carlsbad, CA, USA) using ChamQ Universal SYBR qRT-PCR Master Mix (Vazyme, Nanjing, China). *ACTIN2* was employed as the internal reference gene [79] and relative gene expression levels were analyzed using the 2^–ΔΔCt^ method [80]. The sequences of all primers were documented in Supplemental Table S1.

### Gene cloning and sequence analysis

The full-length cDNA of *VaBAM3* and *VaWRKY65* was amplified from *V. amurensis* leaf cDNA by using specifically designed primers (Supplemental Table S1). The homologous protein sequences from other species were acquired from the NCBI database. The phylogenetic analysis was constructed between VaWRKY65 and all Arabidopsis WRKY proteins via MEGA 7.0 software. Multiple alignment of the amino acid sequences was run in the GENEDOC software.

### Analysis of *VaBAM3* promoter activity

The 1790-bp promoter fragment of *VaBAM3* was fused to pGreenII-0800-LUC vector and transformed into *Agrobacterium tumefaciens* strain GV3101 (pSoup). The fused vector or EV was then transformed into *N. benthamiana* leaves as previously described [81]. After co-culture at 25°C for 2 d, the plants were cultured for 72 h at 4°C. The Dual-Luciferase® Reporter Assay System (Promega, USA) was used to determine the activities of LUC and REN, following the provided instructions. To observe the LUC fluorescence signal, 1 mM D-Luciferin solution was sprayed on the infiltrated leaves with darkness for 5 min. Afterward, the luminescence was detected by a plant living imaging system (Clinx, Shanghai, China).

### Subcellular location analysis

The CDS of *VaBAM3* or *VaWRKY65* without the termination codon was incorporated into the 101LYFP vector. The fused 35S: VaBAM3-YFP vector and 35S: VaWRKY65-YFP vector and 101LYFP EV co-transformed with the nucleus marker (VirD2NLS mCherry) into *N. benthamiana* leaves as previously described. YFP and RFP signals were observed using a confocal laser scanning microscope (Leica TCSSP8, Germany).

### Transcriptional activation activity assay

The full-length and three truncated fragments (F1, F2 and F3) of *VaWRKY*65 were constructed into pGBKT7 vector. All resulting vectors, positive and empty negative control, were introduced into yeast strain AH109 harboring the reporters (ADE2, HIS3, and MEL1), respectively. The transcriptional activation activity of the transformed yeast cells was assessed by spotting the transformants on SD/-Trp, SD/-Trp/-His/-Ade medium or SD/−Trp/-His/-Ade medium supplemented with X-α-gal at 30°C for 3 days.

### Plant transformation and molecular characterization

To obtain transgenic *VaBAM3*- and *VaWRKY65*-overexpressing Arabidopsis (Col-0), the CDS of *VaBAM3* and *VaWRKY65* were fused into pSAK277 vector, then pSAK277-*VaBAM3* and pSAK277-*VaWRKY65* were introduced into *A. tumefaciens* strain GV3101. Arabidopsis floral-dip transformation was carried out as previously described [82].

For grapevine hairy root transformation, overexpressed pSAK277-*VaBAM3* and pSAK277-*VaWRKY65* vectors were constructed. *VaBAM3* and *VaWRKY65* were edited using CRISPR/Cas9 to generate loss-of-function lines as previously described. By the instructions provided by the CRISPR-P Tools software (http://crispr.hzau.edu.cn/cgi-bin/CRISPR2/SCORE), the specific targeted sequence was incorporated into the single guide RNA (sgRNA) construct. *VaBAM3* and *VaWRKY65* targets were imported into pCBC-DT1T2, respectively. The designed sgRNAs were ligated with CRISPR/Cas9 vector pKSE401 [83]. The resulting vectors were transformed into *A. rhizogenes* MSU440. Grapevine hairy root transformation was performed as previously reported with a little modification [84]. Agrobacterium was cultured overnight in liquid TY medium (50 mg/L Kan + 50 mg/L Gent +50 mg/L Rif) until the OD600 reached 0.8. The culture was then incubated with 100 μM acetosyringone (AS) at 28°C for 2 h. The bacterial solution was used to infiltrate cut stems of grapevine seedlings with two buds for 8 min. Following the infiltration, the stems were shifted to 1/2 MS medium and placed in the dark at 25°C for two days. They were then washed with distilled water and transferred to 1/2 MS medium containing 15 mg/L kanamycin, 200 mg/L cefotaxime, and 200 mg/L carbenicillin under standard growth conditions. After four weeks, the identification of positive transgenic lines was conducted through genomic PCR and qRT-PCR analysis. CRISPR/Cas9 genome editing were analyzed by NGS-based Hi-TOM high-throughput sequencing platform to determine the mutation efficiency [92].

For virus-induced gene silencing (VIGS), a nonconserved 324-bp and 350-bp fragment of *VaWRKY65*, *VaBAM3*, and *VaPOD36* were introduced into pTRV2 vector. The fused empty pTRV1, pTRV2, pTRV2-VaWRKY65 vector, pTRV2-VaBAM3, and pTRV2-VaPOD36 vector were separately introduced into *A. tumefaciens* GV3101. The pTRV1 bacterial suspension was combined with pTRV2-VaWRKY65/pTRV2-VaBAM3/pTRV2-VaPOD36 or pTRV2 was infiltrated into 6-week-old tissue culture grapevine plantlets of *V. amurensis* as described previously [85, 86]. The infiltrated plantlets were subsequently rinsed using distilled water and transplanted into soil in a growth chamber under darkness for the first 3 days. And then, qRT-PCR was employed to analyze the expression of target genes in the infiltrated plants.

### Cold tolerance assays

WT and T3 generation *VaBAM3* and *VaWRKY65* transgenic Arabidopsis seedlings were grown on 1/2 MS and maintained in a growth chamber at 22°C for 2 weeks. For Arabidopsis seedlings, 2-week-old seedlings were treated at −6°C for 1 h and transferred to 4°C for 12 h in the dark, followed by 3 days at 22°C. Survival was assessed after treatment. For transgenic grapevine roots, 2-week-old roots were treated at −5°C for 4 h and transferred to 4°C for 12 h in the dark. For VIGS plants, two-month-old VIGS seedlings were subjected to −5°C for 6 h. Samples were obtained at the onset and end of cold treatment for additional physiological analysis and gene expression.

### Physiological measurement

EL was measured using established methods [87]. The MDA, H_2_O_2_ and O_2_^•-^ contents and BAM activities were determined via commercial kits (A003-1 for MDA, A064-1 for H_2_O_2_, A052-1 for O_2_^•-^ and C016-2 for BAM activities, Nanjing Jiancheng Bioengineering Institute, China). The contents of starch and sugar were measured as previously described [51]. DAB and NBT were used for histochemical staining of H_2_O_2_ and O_2_^•-^, respectively, following a previously conducted study [44].

The levels of anthocyanin and proanthocyanidin were determined as previously described [88, 89]. The content of total flavonoids was analyzed using established methods [90].

### RNA-seq analysis

RNA was isolated from the roots of both transgenic and EV control lines using the commercial RNAprep Pure Plant Kit. For each genotype, triplicate samples were analyzed to ensure biological consistency, and RNA integrity and yield were assessed using an Agilent 2100 Bioanalyzer. Subsequently, library construction and sequencing was carried out by Biomarker Company (Beijing, China). High-quality sequences were selected by filtering out poor-quality reads and aligning the remaining reads to the *V. vinifera* reference genome, accessible at the Phytozome database (https://phytozome-next.jgi.doe.gov/info/Vvinifera_v2_1), utilizing HISAT and Bowtie2. The transcript abundance of individual unigenes was quantified using the FPKM method. DEGs were identified based on a fold change threshold of at least 2 and an adjusted *P*-value (FDR) below 0.05. Further functional annotation was conducted through GO term and KEGG pathway enrichment analyses, facilitated by the TBtools software.

### Yeast one-hybrid screening and assays

In the process of library screening, a cDNA library was created using leaves of *V. amurensis* seedlings subjected to cold stress and cloned into the pGADT7-Rec vector. The promoter fragment (−1 to −1790 bp) of *VaBAM3* was fused into the pAbAi vector and then introduced into the *Y1HGold* yeast strain. The screening was carried out according to the protocol using the Matchmaker Gold Yeast One Hybrid Library Kit (Clontech, Mountain View, CA, USA). Every single colony was selected and amplified for sequencing to identify the potential TFs. To confirm the binding of VaWRKY65 and *VaBAM3/VaPOD36* promoter, the full-length CDS of *VaWRKY65* was fused into the pGADT7 vector to generate VaWRKY65-pGADT7 prey vector. The promoter fragments of *VaBAM3* and *VaPOD36* containing W-box were inserted into the pAbAi vector, while the W-box (TGAC) was replaced by TTTT to create mutational bait. The yeast *Y1HGold* strains containing prey and bait/mutant bait were produced. The yeast clones that had undergone a transformation were plated on the selective medium (SD/-Leu/-Ura) with or without 200 ng/ml AbA for 3 days.

### Dual-LUC assay

The full-length CDS of *VaWRKY65* was inserted into the pGreen II 62-SK vector to generate an effector. The original and mutated promoter sequences of *VaBAM3* and *VaPOD36* were cloned into pGreen II 0800-LUC vector to act as reporters. All effectors and reporters were transiently co-expressed in *N. benthamiana* leaves as previous report [44]. The LUC activities were evaluated using Dual-Luciferase Reporter Assay Kit (Cat. E1910, Promega, USA). The infiltrated leaves were sprayed with D-Luciferin solution (1 mM), followed by darkness for 5 min. Then, the LUC fluorescence signal was observed by NightSHADE LB 985 (Berthold, Germany).

### EMSA assay

The full-length CDS of *VaWRKY65* was cloned into the pET-28a vector containing a His tag, and the fused plasmid was transformed into *Escherichia coli* BL21 (DE3) strain to construct the His-VaWRKY65 fusion protein. The recombinant His-VaWRKY65 fusion protein was induced by 0.5 mM IPTG at 30°C for 4 h and purified by using the Ni-NTA agarose. The DNA probes containing either W-box or mutated elements were synthesized with biotin-label by Tsingke Biological Technology (Beijing, China). An unlabeled probe with the same sequence was used as the competitor. The EMSA was performed using the Chemiluminescent EMSA kit (Beyotime, Shanghai, China) as previously described [91].

### ChIP-qPCR assay

Transient expression of *VaWRKY65* in grapevine was carried as previous described [16, 44]. The grapevine leaves agroinfiltrated by 35S:FLAG and VaWRKY65-FLAG were cross-link protein-DNA in 1% (w/v) formaldehyde. After fragmentation by sonication (Bioruptor plus, Belgium), the chromatin was immunoprecipitated with anti-FLAG antibody (F1804; Sigma, USA). The purified protein–DNA was used for qPCR by specific primers (Table S1).

### Statistical analysis

All experiments were repeated at least three times. All data were presented as the mean ± SE (standard error). Statistical differences were analyzed using one-way analysis of variance (ANOVA, Waller-Duncan) in SPSS, with a significance level of *P* < 0.05 (^*^) and *P* < 0.01 (^**^).

## Supplementary Material

Web_Material_uhae367

## Data Availability

The leaves of *V. amurensis* under chilling stress RNA-seq data were deposited into NCBI GEO database with the accession number PRJNA934965.
