# Correction to: *VaWRKY65* contributes to cold tolerance through dual requaltion of soluble sugar accumulation and reactive oxygen species scavenging in *Vitis amurensis*

**DOI:** 10.1093/hr/uhag317

**Published:** 2026-07-29

This is a correction to: Lin Meng, Huimin Zhou, Lisha Tan, Qingyun Li, Yujun Hou, Wenjuan Li, Subash Kafle, Ju Liang, Rishi Aryal, Zhenchang Liang, Haiping Xin, *VaWEKY65* contributes to cold tolerance through dual regu;ation of soluble sugar accumulation and reactive oxygen species scavenging in *Vitis amurensis*, *Horticulture Research*, Volume 12, Issue 4, April 2025, uhae367, https://doi.org/10.1093/hr/uhae367

In the originally published version of this manuscript, under the ‘Cold induction of VaBAM3’, there was an error in the description of the peak time.

The sentence concerned should read: “qRT-PCR analysis suggested that VaBAM3 expression increased progressively under low-temperature treatment, peaking at 48 h (Fig. 1B)” instead of: “qRT-PCR analysis suggested that VaBAM3 expression increased progressively under low-temperature treatment, peaking at 24 h (Fig. 1B)”.

This error has been corrected only in this correction notice to preserve the version of record.

