# Supplementary material for: VaWRKY65 contributes to cold tolerance through dual regulation of soluble sugar accumulation and reactive oxygen species scavenging in Vitis amurensis
Source: Hortic Res. 2025 Jan 3;12(4):uhae367. doi: 10.1093/hr/uhae367 (PMC11896968; doi:10.1093/hr/uhae367)
Supplement: Web_Material_uhae367 [file web_material_uhae367.zip › Supplemental Figure files.docx]

**
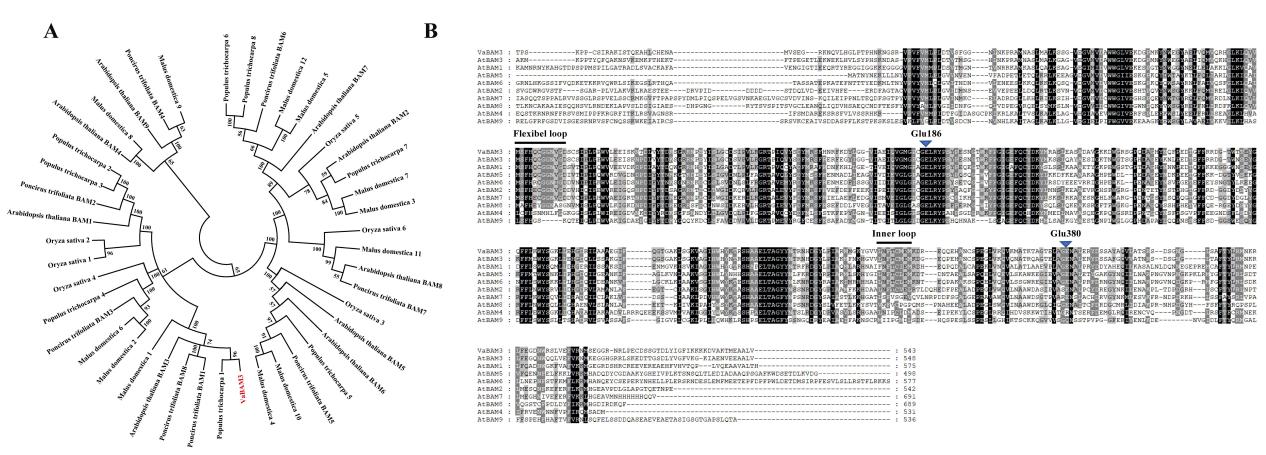
**

**Figure S1.** Phylogenetic analysis and multiple alignments of VaBAM3 with other plant species. **A** A phylogenetic tree was constructed based on the amino acid sequences of VaBAM3 with BAM gene from *Arabidopsis thaliana*, *Malus x domestica*, *Populus trichocarpa*, *Poncirus trifoliata*, and *Oryza sativa* by using MEGA 7.0 software based on the neighbour-joining method and bootstrap analysis with 1000 replications. **B** Multiple alignments were performed with GENEDOC software using the conserved domain of VaBAM3 with *Arabidopsis* orthologs, AtBAM1–9. The stars labled as * indicates C2H2 motif site. Substrate binding sites and two catalytic sites (Glu186 and Glu380) are marked with large blue arrow- heads. The residues forming flexible loop and inner loop are indicated with solid lines above the sequences.

**
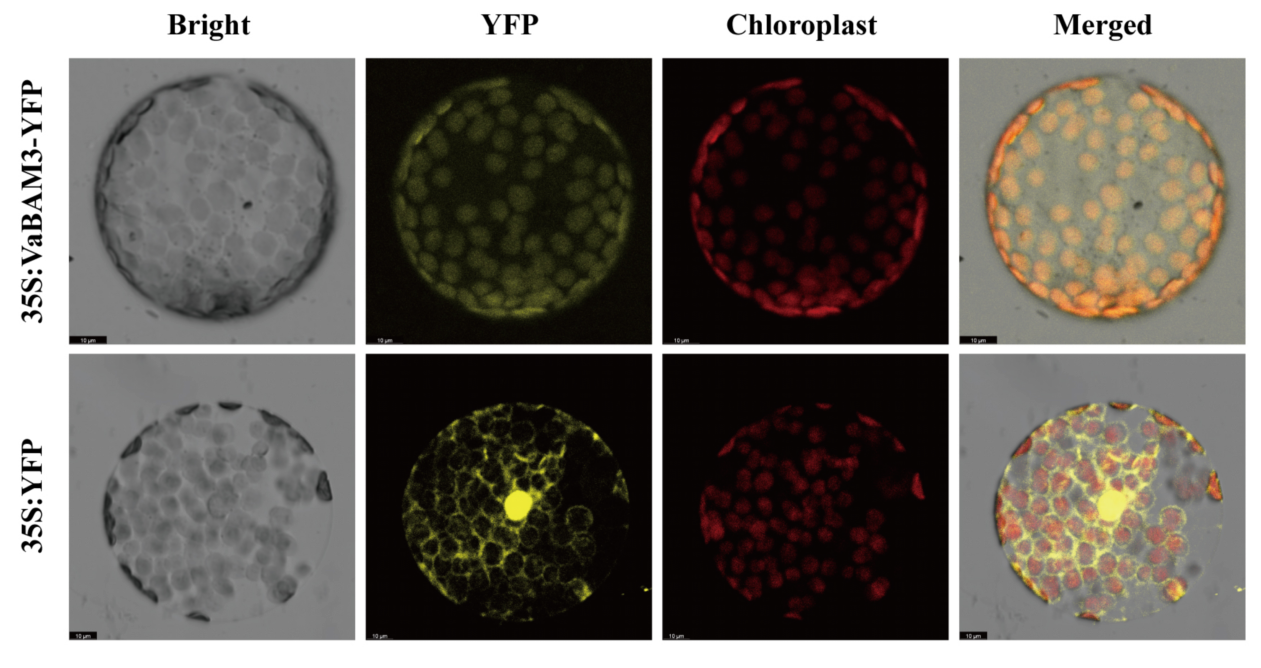
Figure S2.** Subcellular localization of VaBAM3 protein. The tobacco leaves (*N. benthamiana*) were infiltrated with the fusion construct (35S: VaBAM3-YFP) or an empty vector (35S: YFP). Confocal images display yellow fluorescence signals for YFP and red fluorescence signals for chloroplasts in protoplasts. Scale bars=10 µm.


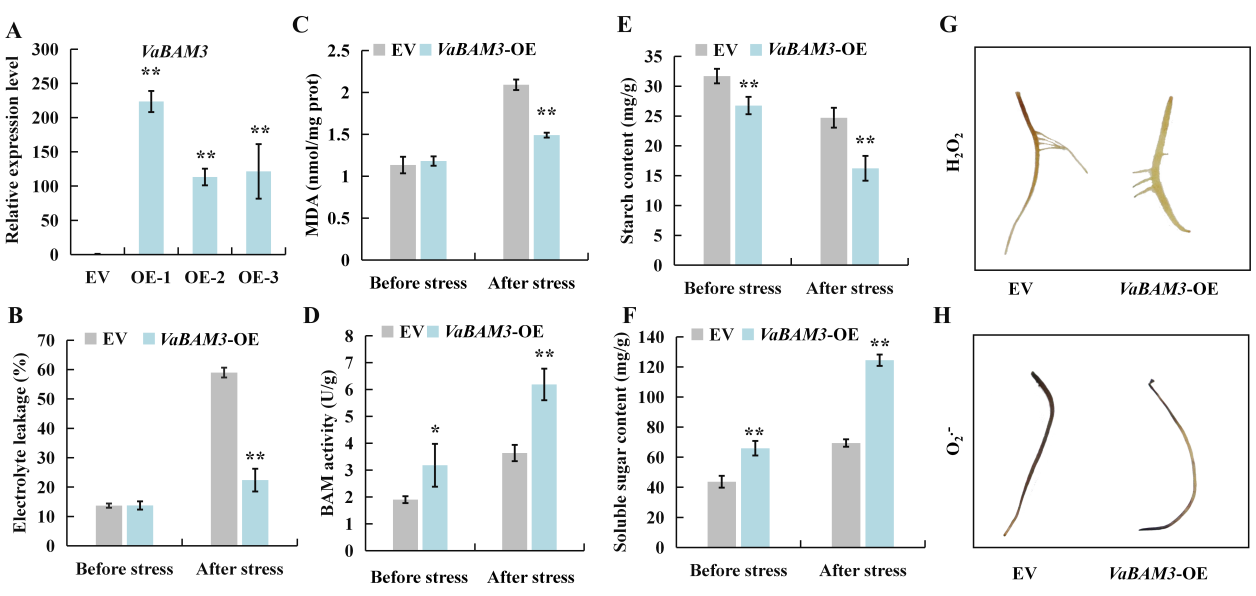


**Figure S3.** Overexpression of *VaBAM3* confers cold tolerance in grapevine roots. **A** qRT-PCR analysis verified the expression level of *VaBAM3* in EV and overexpression grapevine roots. **B**-**F** Electrolyte leakage (**B**), MDA (**C**), BAM activity (**D**), starch content (**E**) and soluble sugar content (**F**) in EV and overexpression grapevine roots before and after cold treatment. **G**-**H** *In situ* detection of H_2_O_2_ (**G**) and O_2_^•-^ (**H**) in EV and overexpression grapevine roots after cold treatment, as revealed by histochemical staining with DAB and NBT, respectively. Error bars represent ± SE (n=3). Asterisks indicate significant difference between EV and overexpression grapevine roots under same conditions (*P < 0.05, **P < 0.01).

**
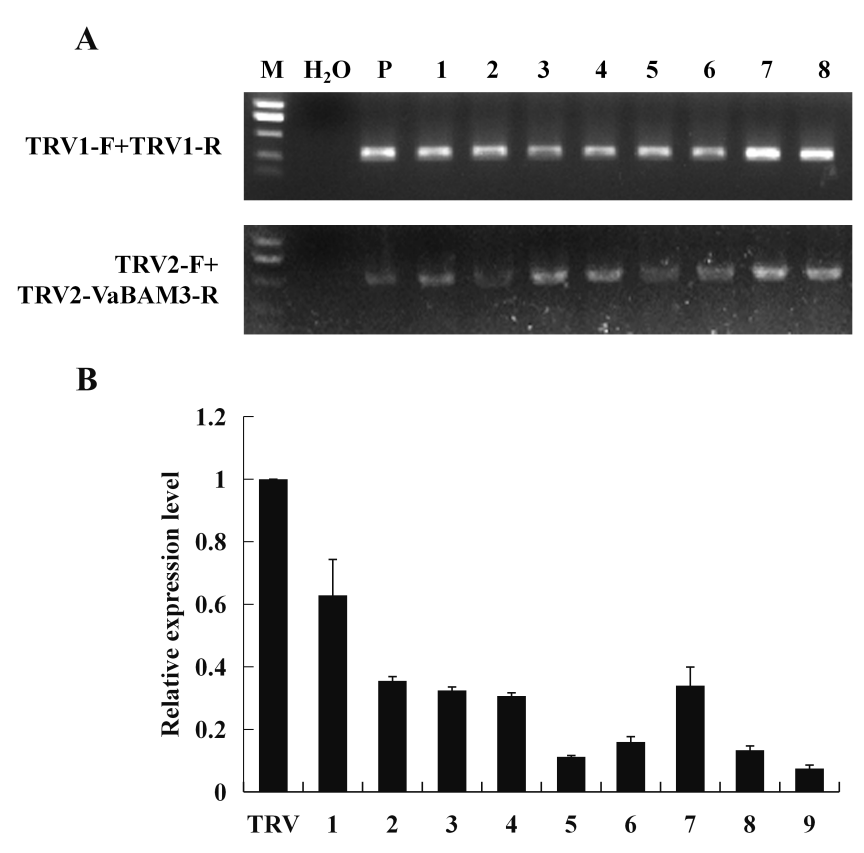
**

**Figure S4.** Molecular identification of TRV-*VaBAM3* VIGS plants. **A** The transformed vectors were exampled by PCR using genomic DNA samples. M: DNA marker; P, plasimid. **B** qRT-PCR was used to analyze the *VaBAM3* expression of TRV2-*VaBAM3* plants, using. *Actin* was used as an internal control. Error bars indicate ± SE (n = 3).


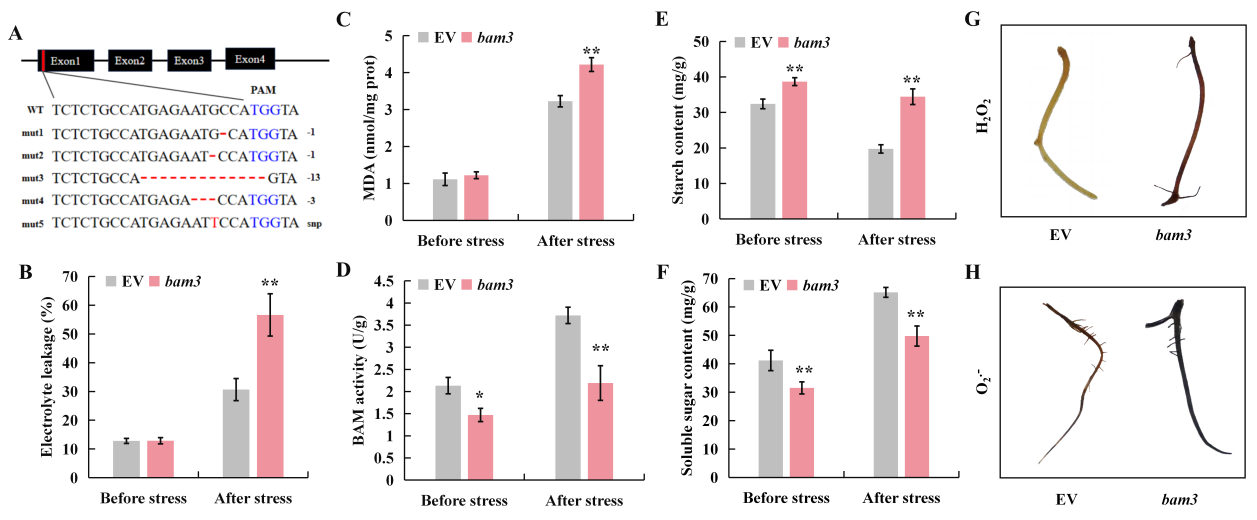


**Figure S5.** Knockout of *VaBAM3* decreases cold tolerance in grapevine roots. **A** Sequencing results of *VaBAM3* knockout mutant (*bam3*). Minus (-) signs indicate the number of nucleotides deleted and inserted at target site. **B**-**F** Electrolyte leakage (**B**), MDA (**C**), BAM activity (**D**), starch content (**E**) and soluble sugar content (**F**) in EV and mutant *bam3* grapevine roots before and after cold treatment. **G**-**H** *In situ* detection of H_2_O_2_ (**G**) and O_2_^•-^ (**H**) in EV and mutant *bam3* grapevine roots after cold treatment, as revealed by histochemical staining with DAB and NBT, respectively. Error bars represent ± SE (n=3). Asterisks indicate significant difference between EV and mutant grapevine roots under same conditions (*P < 0.05, **P < 0.01).


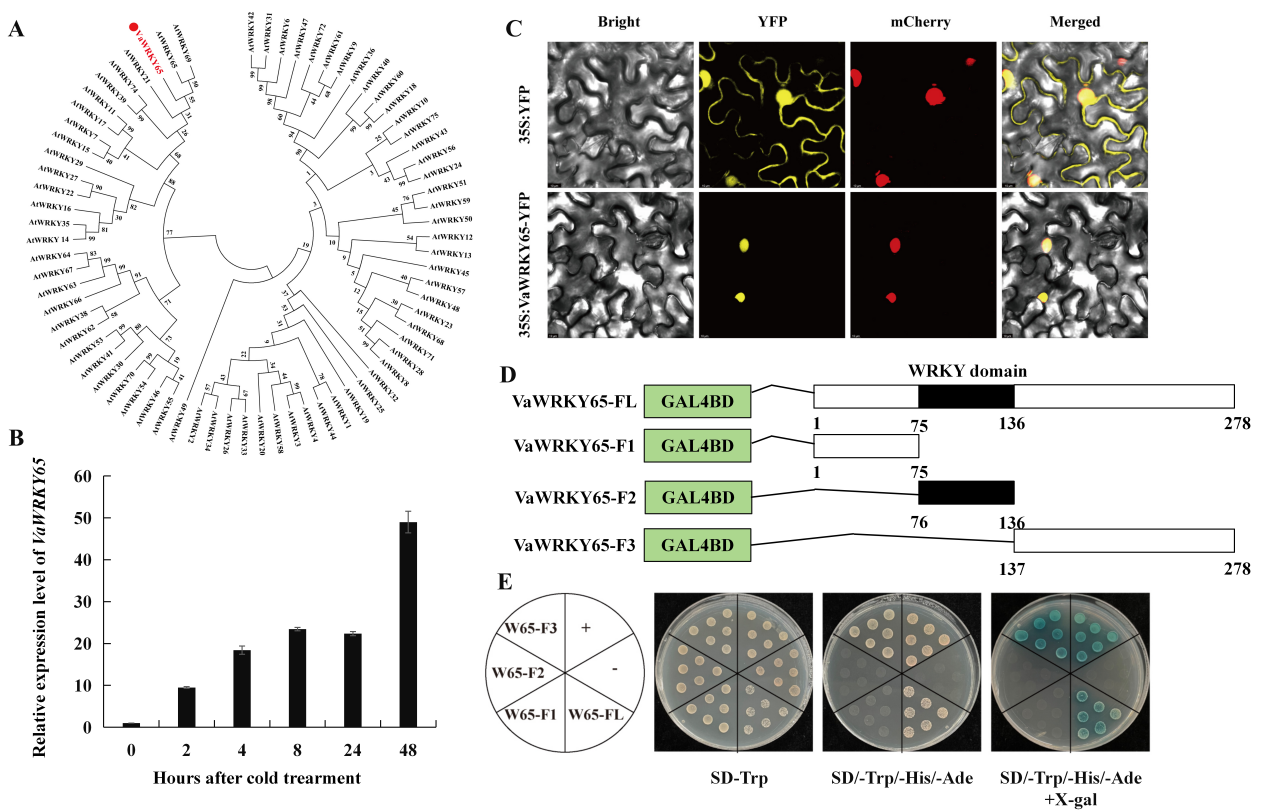


**Figure S6.** Characterization of *VaWRKY65* under cold stress in *Vitis amurensis.* **A** A phylogenetic tree was constructed based on the amino acid sequences of VaWRKY65 with 73 annotated WRKY TFs from *Arabidopsis thaliana* by using MEGA 7.0 software based on the neighbour joining method and bootstrap analysis with 1000 replications. **B** Relative expression levels of *VaWRKY65* under cold treatment. Error bars represent ± SE (n=3). **C** Subcellular location of VaWRKY65. The fusion construct 35S: VaWRKY65-YFP or 35S: YFP empty vector was co-transformed with *VirD2NLS-*mCherry (a nucleus marker) in tobacco (*N. benthamiana*) leaves. Confocal microscopic images showed bright filed, yellow (for YFP), red (for mCherry) fluorescence signals in epidermal cells. Scale bars=10 μm. **D** Schematic diagrams of the full length (FL) and three truncated fragments (F1, F2 and F3) of VaWRKY65 used for constructing vectors. All constructed vectors were introduced in pGBKT7 vector with GAL4 DBD domain. The numbers below the bars indicate the positions of amino acid. **E** Transcription activity assay. Growth of yeast cell (AH109 strain) transformed with various constructed vectors on SD/-Trp and SD/-Trp/-His/-Ade with or without X-α-gal selective medium. Transformation of pGBKT7-p53 and pGBKT7 vector was used as a positive and negative control, respectively.


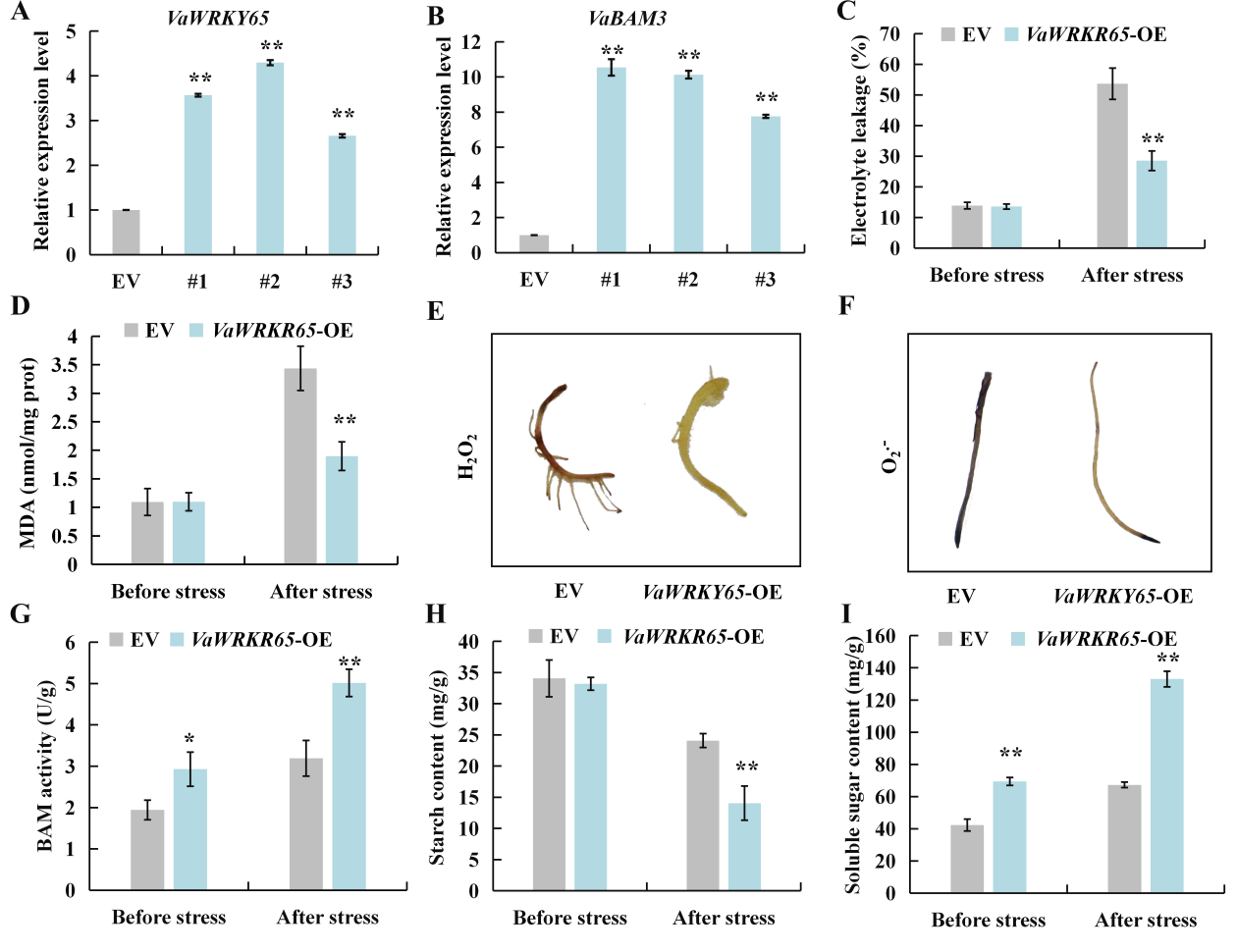


**Figure S7.** Overexpression of *VaWRKY65* confers cold tolerance in grapevine roots. **A**-**B** qRT-PCR analysis verified the expression levels of *VaWRKY65* (**A**) and *VaBAM3* (**B**) in EV and overexpression grapevine roots. **C**-**D** Electrolyte leakage (**C**) and MDA content (**D**) in EV and overexpression grapevine roots before and after cold treatment. **E**-**F** *In situ* detection of H_2_O_2_ (**E**) and O_2_^•-^ (**F**) in EV and overexpression grapevine roots after cold treatment, as revealed by histochemical staining with DAB and NBT, respectively. **G**-**I** BAM activity (**G**), starch content (**H**) and soluble sugar content (**I**) in EV and overexpression grapevine roots before and after cold treatment. Error bars represent ± SE (n=3). Asterisks indicate significant difference between EV and overexpression grapevine roots under same conditions (*P < 0.05, **P < 0.01).


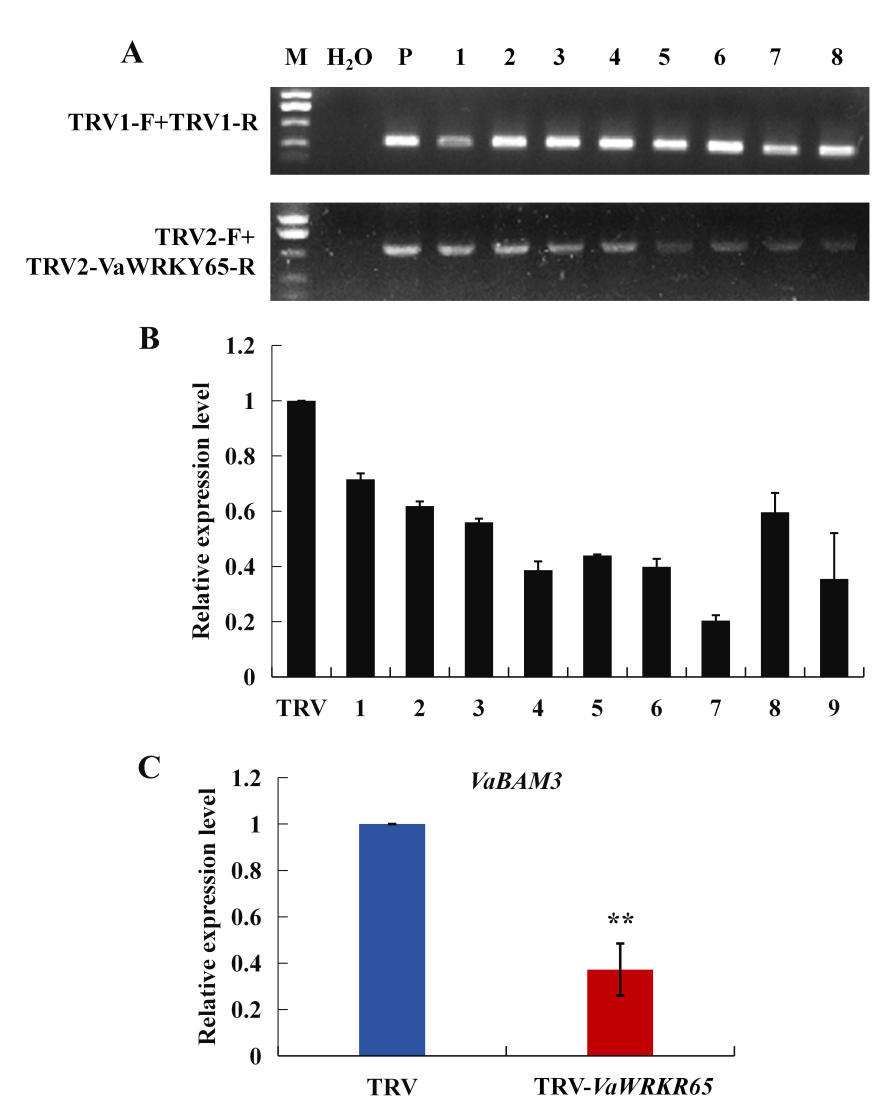


**Figure S8.** Molecular identification of TRV-*VaWRKY65* VIGS plants. **A** The transformed vectors were exampled by PCR using genomic DNA samples. M: DNA marker; P, plasimid. **B** qRT-PCR was used to analyze the *VaWRKY65* expression of TRV2-*VaWRKY65* plants, using. *Actin* was used as an internal control. Error bars indicate ± SE (n = 3). **C** qRT-PCR analysis verified the expression levels of *VaBAM3* in TRV and TRV-*VaWRKY65* VIGS plants.


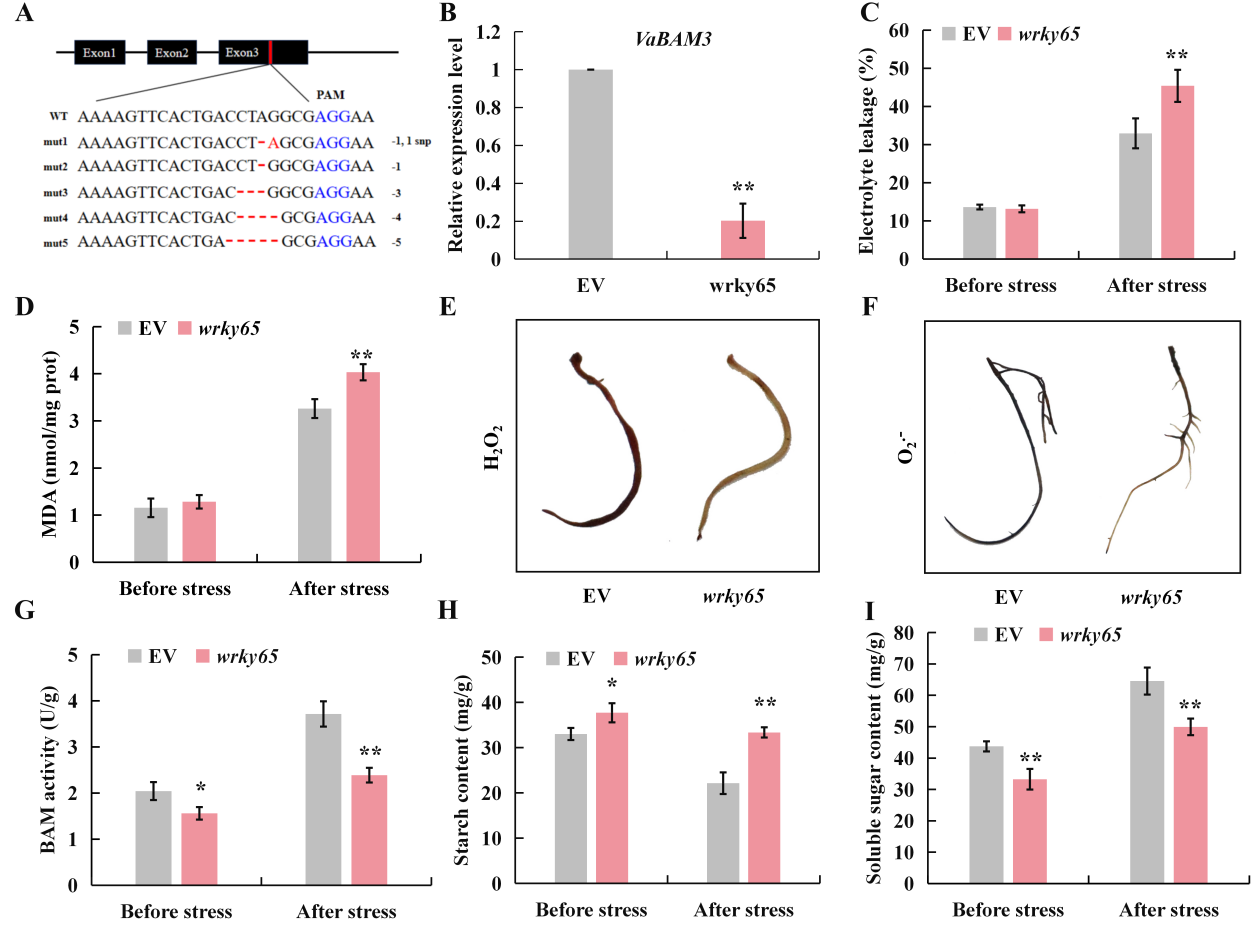


**Figure S9.** Knockout of *VaWRKY65* decreases cold tolerance in grapevine roots.

**A** Sequences results of *VaWRY65* knockout mutant (*wrky65*). Minus (-) signs indicate the number of nucleotides deleted and inserted at target site. **B** qRT-PCR analysis verified the expression levels of *VaBAM3* in EV and mutant grapevine roots. **C**-**D** Electrolyte leakage (**C**) and MDA (**D**) in EV and mutant *wrky65* grapevine roots before and after cold treatment. **E**-**F** *In situ* detection of H_2_O_2_ (**E**) and O_2_^•-^ (**F**) in EV and mutant grapevine roots after cold treatment, as revealed by histochemical staining with DAB and NBT, respectively. **G**-**I** BAM activity (**G**), starch content (**H**) and soluble sugar content (**I**) in EV and mutant grapevine roots before and after cold treatment. Error bars represent ± SE (n=3). Asterisks indicate significant difference between EV and mutant grapevine roots under same conditions (*P < 0.05, **P < 0.01).

**
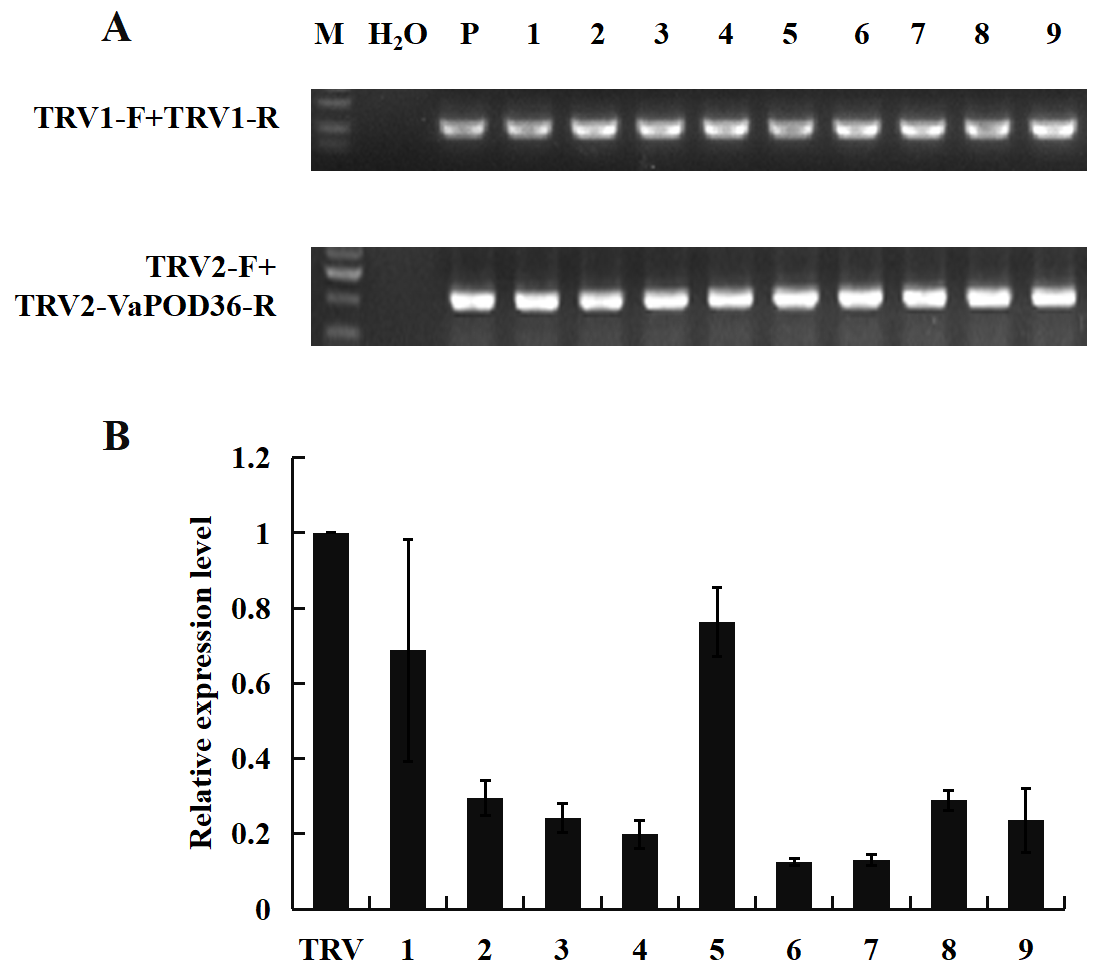
**

**Figure S10.** Molecular identification of TRV-*VaPOD36* VIGS plants. **A** The transformed vectors were exampled by PCR using genomic DNA samples. M: DNA marker; P, plasimid. **B** qRT-PCR was used to analyze the *VaPOD36* expression of TRV2-*VaPOD36* plants, using. *Actin* was used as an internal control. Error bars indicate ± SE (n = 3).


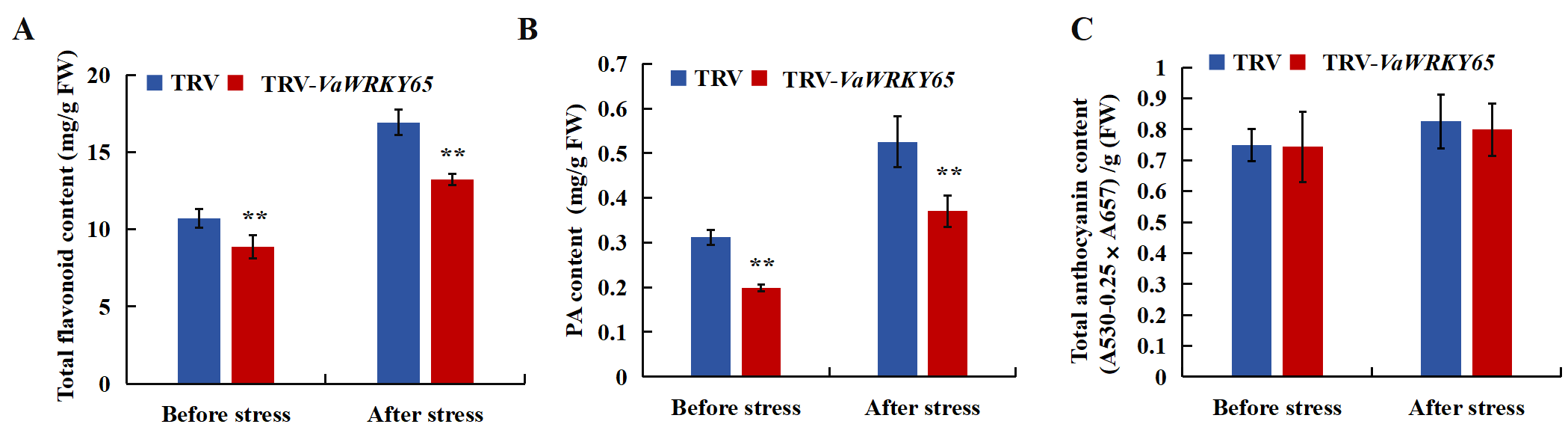


**Figure S11.** *VaWRKY65* promotes synthesis of various metabolites. **A**-**C** The total flavonoid (**A**), proanthocyanidin (PA) (**B**) and total anthocyanin (**C**) contents in the TRV-*VaWRK65* and TRV control plants.


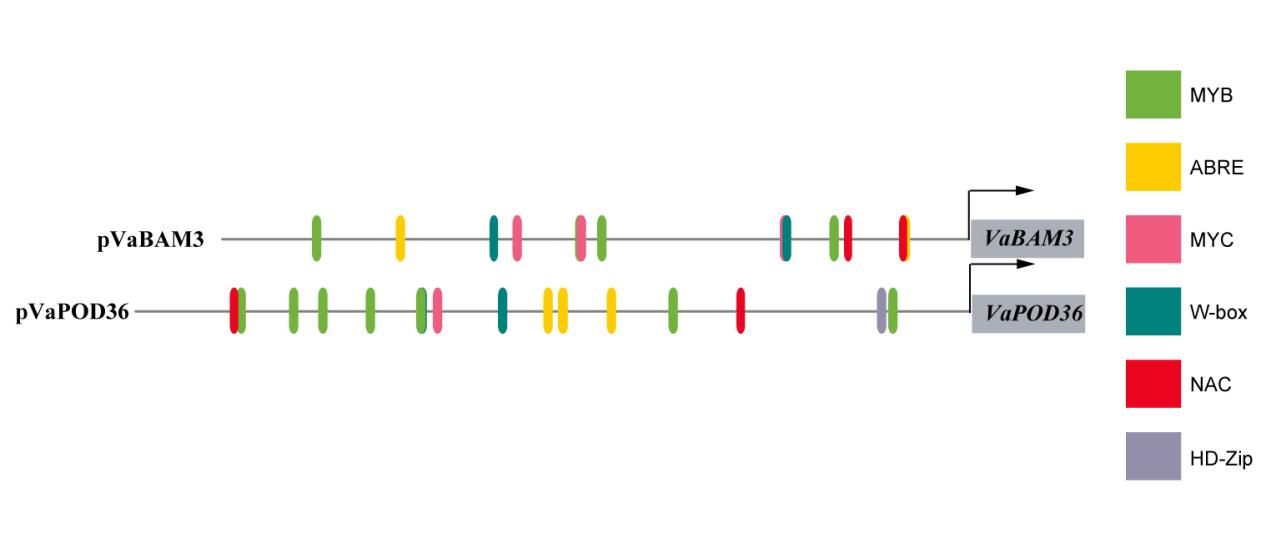


**Figure S12.** Predicted cis-elements in the promoter regions of *VaBAM3* and *VaPOD36*.
